# Patient satisfaction and willingness as indicators for patient perspectives toward trainee involvement: a systematic review

**DOI:** 10.1186/s12909-025-08310-4

**Published:** 2025-12-22

**Authors:** Sarah de Bever, Mana Nasori, Helianne Wisse, Mechteld Visser, Nynke van Dijk

**Affiliations:** 1 Department of General Practice, Quality of Care and Personalized Medicine, Amsterdam UMC location AMC, Amsterdam Public Health, Meibergdreef 9, Amsterdam, the Netherlands; 2 Sports and Physical Activity, Amsterdam University of Applied Science – faculty of Health, Centre of Expertise Urban Vitality, Amsterdam, the Netherlands

**Keywords:** Systematic review, Trainee, Register, Ambulant care, General practice, Patient, Opinion, Satisfaction, Willingness

## Abstract

**Purpose:**

Medical trainees learn from treating patients. Patients’ refusal of trainees as caregivers influences the types of patients who trainees see and thereby their learning. This is likely to be even more pronounced in ambulatory care facilities, where patients have more say in the choice of doctor. Understanding patients’ perspectives on trainee involvement in their care may provide useful insights into how to enhance their acceptance of trainees. Therefore, we conducted a systematic review of the literature, focussing on patients’ perspectives on trainee involvement in their care and factors affecting these perspectives in the ambulatory care setting.

**Methods:**

We searched four databases (EMBASE, Medline, ERIC, and PsychInfo) from the start of each database until April 2024, combining keywords for ambulatory care settings, residency, and patient attitudes. Two researchers independently performed the review process.

**Results:**

A total of 8735 studies were identified, 38 of which were included. Most studies had a survey design. The mean MERSQI score was 8.7, indicating low quality. Studies have reported various outcomes, of which satisfaction and willingness with trainee involvement are overarching themes. Most studies reported notably high satisfaction rates, often exceeding 90%, and more than 70% of patients reported trainee involvement. However, acceptance rates decline rapidly when tasks or reasons for consultation are more complicated or when patients have long-standing illnesses. Patients were also less satisfied with trainees’ interpersonal competencies than were fully trained doctors. A positive relationship was found between previous treatment by a trainee and patients’ willingness to consult a trainee.

**Conclusion:**

Patients generally report high levels of satisfaction and willingness to engage with trainees, particularly in straightforward consultations or when prior contact exists. Nonetheless, lower ratings of trainees’ interpersonal competencies and reduced willingness in complex or chronic cases underscore the need for improved communication training and greater continuity in trainee‒patient interactions.

**Supplementary Information:**

The online version contains supplementary material available at 10.1186/s12909-025-08310-4.

## Introduction

Active participation in routine patient care is essential for the development of competencies in medical trainees [1, 2]. However, patients are not obligated to accept a learner and have the autonomy to decide whether they wish to involve a trainee in their care. From the trainee’s perspective, a patient’s refusal represents a missed learning opportunity. This is particularly unfortunate, as research has shown that trainees are already involved in only a limited number of potential learning opportunities [3]. While this limitation can be attributed to a range of factors, including trainee engagement [3–5] and the resources available at the training site [6, 7], patient willingness to allow a trainee’s involvement is undeniably a key factor. This is likely to be even more significant in ambulatory care settings, where patients typically have greater freedom of choice in selecting their physician but also experience continuity of care and the opportunity to develop an established therapeutic relationship, in contrast to the inpatient setting. Understanding the reasons behind patients’ acceptance or refusal can help enhance learning opportunities for trainees.

Previous reviews on this topic have focused primarily on medical students [8] or have been limited to a single specialty [9]. These reviews report that most patients are satisfied with the involvement of medical students, although their willingness varies on the basis of the perceived risk and/or the sensitivity of the patient’s reason for consultation [8, 9]. While these reviews offer valuable insights, we still lack insight into patients’ perspectives on the involvement of medical trainees (graduated doctors who are in training to become specialists) in ambulatory care settings.

Therefore, we conducted a systematic review with two primary aims: (1) to describe and summarize patients’ perspectives on trainee involvement in their care and (2) to identify the factors that influence these perspectives.

## Methods

### Data sources and searches

For this systematic review, we searched MEDLINE, Embase, PsychINFO, and the Education Resource Information Centre (ERIC). The databases were searched from the start of the database until April 2024. The keywords were determined on the basis of previously identified relevant articles [10–13] in consultation with the clinical librarian. The databases were searched by combining keywords for ‘ambulatory care setting’, ‘medical residency’, and ‘patient attitudes’. The complete search string for each database can be found in Appendix I.

### Inclusion and exclusion criteria

Studies were included if they were written in English or Dutch and if they described attitudes, opinions, or perspectives of patients regarding trainee involvement in their care in an ambulatory care setting. We defined a “trainee” as a graduate medical doctor who is in training to become a specialist (including general practitioners). Trainees of all levels were included. We did not specify the degree of involvement of the trainee; thus, involvement could range from independent practice to being an observer. The ambulatory care setting was defined as an outpatient clinic of a hospital or a general practice. There were no restrictions regarding patient age, sex, or condition.

Studies were excluded if there were no clear findings on attitudes, opinions, or perspectives towards the trainee; for example, studies of patient views on waiting times or knowledge about the role of the trainee were excluded. Studies on patient attitudes towards trainees in a hospital setting (e.g., wards) were also excluded. Other reasons for exclusion were other types of education (e.g., dental or undergraduate) or nonresearch formats (e.g., commentaries or letters to the editor).

### Study selection

The studies were assessed independently on title and abstract by pairs of two authors (SdB and HW, SdB and MN, SdB and NM, or SdB and MV). In cases of disagreement, a decision was reached by negotiated consensus. In cases of persistent disagreement or doubt, the article was included for full-text screening. For all abstracts that appeared to be eligible, the full texts were retrieved and screened independently by two authors. In case of disagreement, a decision was reached through negotiated consensus. If an agreement could not be reached, a third researcher (MV or MN) was consulted to make a final decision. Additionally, the reference lists of all studies included were manually searched for additional studies.

### Data extraction

Data on the study setting, design, objectives, participant characteristics, sample size, trainee level, level of supervision, attitudes towards trainees, and reported effects of various factors related to attitudes were extracted via SdB. MN and NvD checked the extracted results for accuracy by comparing them with the original papers. During the extraction process, findings were regularly discussed with other members of the research team.

### Quality assessment

The Medical Education Research Study Quality Instrument (MERSQI) was used to assess the quality of the quantitative studies [14]. The MERSQI consists of 6 domains, such as “Study design” and “Validity evidence of evaluation instrument”, on which a score of a maximum of three points can be obtained, resulting in a total maximum score of 18. For the qualitative studies, we used The COnsolidated criteria for REporting Qualitative research (COREQ) instrument to describe the quality of reporting [15]. Although the COREQ is designed to assess the quality of reporting, the reporting criteria are evidently also relevant to quality. By assessing not only whether the authors reported on a specific criterion but also what they reported, a reasonably good impression of the quality of the study can be obtained. MV and SdB independently assessed six studies, after which a comparison was made. No differences were found, after which SdB completed the quality assessment for the remaining studies. In case of doubt, MV was consulted for guidance. MN assessed the article of de Bever et al., because SdB was the author and was therefore not involved in the assessment of that study.

## Results

### Search results

**Fig. 1:**
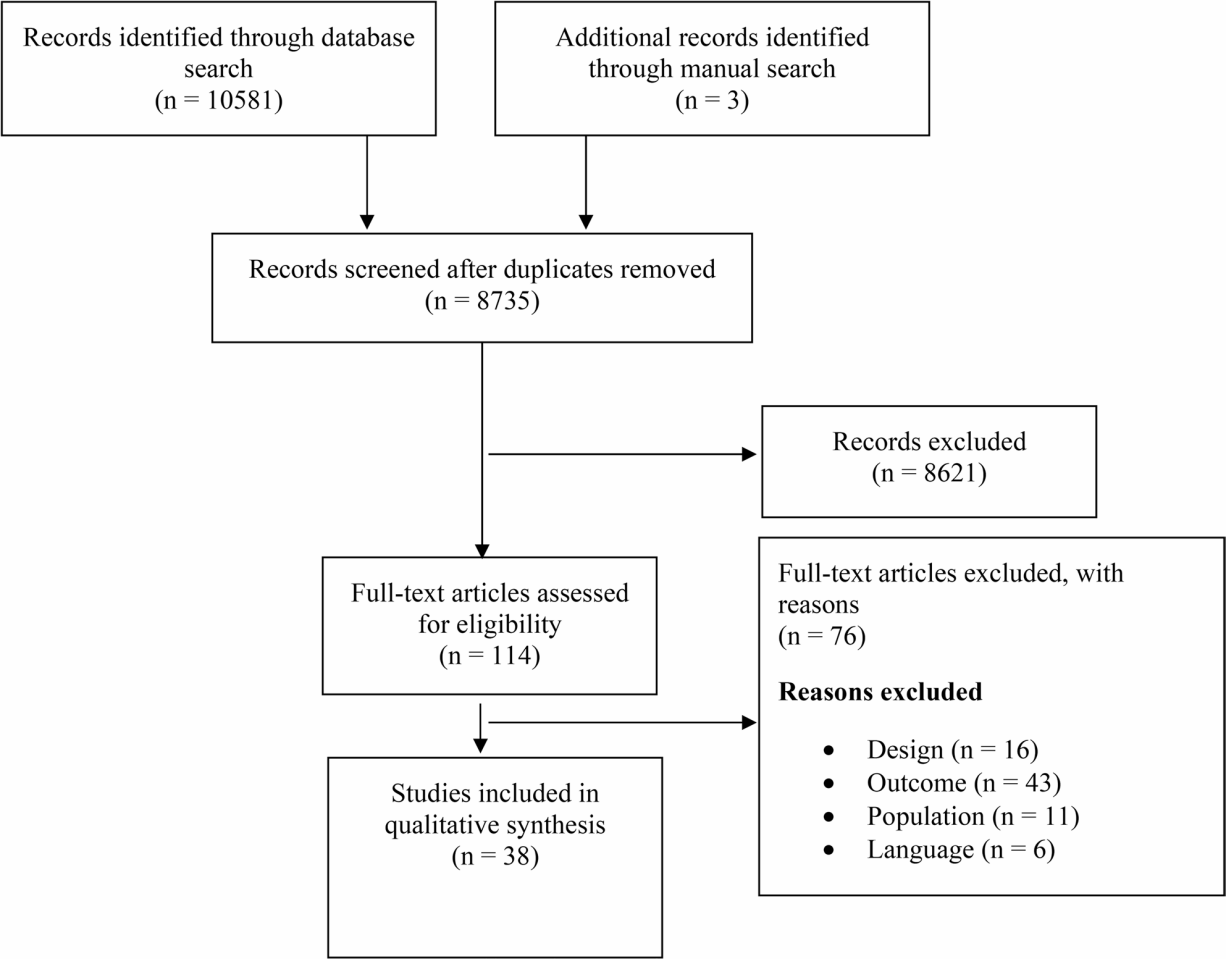
Flowchart of study selection

In total, 8735 studies were identified. After screening the titles and abstracts, 114 articles remained for full-text review. A total of 38 studies met the inclusion criteria and were selected for this review (Fig. 1). 

### Study characteristics

The included studies were published between 1977 and 2023. Except for two qualitative interview studies [10, 16], all studies were survey studies. The studies covered eight medical specialties, of which general practice/family medicine was most prevalent (*n* = 20). The participants were patients who visited a clinic or practice during a specified period, varying between one day and one year. Most studies did not have inclusion or exclusion criteria for their participants, except for the studies by Bonney et al., and Haider et al., Boney et al., included only patients above 60 years of age [10–12, 17], and Haider et al., included those between 20 and 40 years of age [18]. The participation rates varied between 23.7% [19] and 94.7% [13], and eleven studies did not report a participation rate [18, 20–29]. Eighteen studies provided details about the trainee, twelve reported on the trainee level of training [16, 18, 19, 25, 27, 29–35], and eight reported on the level of supervision a trainee received [13, 18, 21, 22, 27, 34, 36, 37]. Appendix II provides an overview.

### Quality, validity and instruments used

The quality of the studies included was low. The mean (SD) total MERSQI score was 8.5 (1.5), with a range of 6–11 (see Appendix III, section A, for the MERSQI scores). The COREQ reports for the qualitative studies are presented in Appendix III (section B).

### Patient satisfaction and willingness

In most of the included studies, patient satisfaction and willingness were the primary reported outcome measures; therefore, we decided to present our results around these two indicators from the patient’s perspective. However, these outcomes were operationalized in various ways, with considerable variation in their level of specificity. Studies reported outcomes ranging from general measures—such as overall satisfaction with the resident or general willingness to be seen—to more specific assessments, such as satisfaction with particular behaviors (e.g., empathy and humaneness) and communication skills (e.g., listening and providing explanations), or willingness to be seen by a resident in specific clinical scenarios. We have grouped this wide range of outcomes under two overarching themes: *patient satisfaction* and *willingness.* In the following sections, we first report on satisfaction and willingness, followed by an exploration of the factors influencing these outcomes.

### Satisfaction

In total, twenty-one studies [11, 12, 17, 19–22, 25, 27–30, 32–35, 37–41] reported the satisfaction of patients with trainee involvement in their care. Eight [11–13, 24, 30, 36, 37, 42] reported on patient satisfaction with trainees, and seventeen [11, 19–22, 25, 27–30, 32–35, 39–41] compared the satisfaction rates of patients seen by trainees and those seen by faculty (defined as doctors who completed their medical specialty training irrespective of medical specialism). The studies that reported overall satisfaction indicated that most patients are very satisfied with trainees, with satisfaction rates varying between 74.3% and 99.5% [11–13, 30, 36, 37, 42]. Patients who find trainees attentive listeners are more likely to have a good experience with the trainee [24].

In the following paragraphs, we report patient satisfaction with trainees compared with faculty. The outcomes included overall satisfaction, satisfaction with the quality of care, satisfaction with communication skills, and satisfaction with doctor behavior. Table 1 provides an overview of the results.

**Table 1:** Patient satisfaction with care received from trainees vs. care received from faculty, divided into overall satisfaction, quality of care, specific skills and behavior

Study	Outcomes	Results
Trainee vs. faculty – Overall satisfaction with received care		
* Brahmania et al., 2015*	Satisfaction with the received care of patients seen by trainees and attending physician vs. patients seen by faculty alone	No statistically significant effect in overall satisfaction OR 0.9 (95% CI: 0.06–13.7)
* Cao & Chen et al., 2018*	Satisfaction with the received care of patients seen with trainee involvement vs. patients seen without trainee involvement	No statistically significant difference in overall satisfaction scores M (SD nr*): 4.9 (alone) vs. 4.8 (trainee) (scale 1–5) (*p* = 0.8)
* Jaturapatporn et Dellowl 2007*	Satisfaction with the received care of patients seen by trainees vs. general doctor vs. faculty	No statistically significant difference in overall satisfaction M (SD nr): faculty 80.7 (14.4)/ general doctor 79.7 (141)/trainees 80.9 (15.5) (*p* = 0.4)
* Norris et Flaherty 1993*	Satisfaction with the received care of patients seen by a trainee alone vs. faculty alone vs. trainee and faculty	No difference in mean satisfaction M (SD nr) : faculty 4.3/ faculty + trainee 4.1/ trainee 3.6 (scale 1–5) (*p* > 0.05)
* Thornett et al, 2001*	Satisfaction with the received care of patients seen by trainees vs. patients seen by GPs (faculty)	Patients seen by a GP are more satisfied than those seen by a trainee M (SD nr): 75.6 (GP trainee) vs. 82.2 (GP) (scale 1-100) (*p* = 0.022)
* Yancy et al., 2001*	Satisfaction with the received care of patients seen by a trainee vs. patients seen by faculty (split on site: university clinic (UC) or veteran affairs (VA) clinic)	Patients seen by faculty at the UC are more likely to be perfectly satisfied than those seen by trainees; not significantly different at the VA clinic UC: OR 3.9 (95% CI:1.2–12.5) VA: OR 0.95 (95% CI: 0.4–20.1)
* Sherbuk et al., 2019*	Overall reported satisfaction of patients seen by a trainee or a HIV-specialist (faculty)	No statistically significant difference M (SD nr) : HIV specialist 9.6 vs. trainee 9.7 (scale 1–10) (*p* = 0.71)
* Rodrigues et al., 2023*	Percentage that answered complete to the question: ‘Did this visit meet your needs?’ (*n* = 1381)	No statistically difference Faculty 68% vs. trainee 62% (ns)
Trainee vs. faculty – Satisfaction with quality of care		
* Allen et al., 1981*	Agreement with statement: “The trainee gives the same quality of care as the usual GP (faculty)” (n = nr)	61.3% agreed GP trainees to give the same quality of care as GPs ; 18.2% did not agree
* Blanchard et al., 1977*	Perceived quality of care rating of patients seen by a trainee vs. patients seen by faculty (n = nr)	40% could see no difference in quality of care between trainees and faculty
* Rodney et al., 1986*	Perceived quality of care of patients seen by a trainee vs. patients seen by faculty	No statistically significant difference M (SD nr): faculty 80.5 vs. trainee 68.7; (*p* = 0.09)
Trainee vs. faculty – Satisfaction with communication skills		
* Bradley et al., 1981 l*	Percentage of patients seen by a trainee (*n* = 42) vs. those seen by faculty (*n* = 206) agreeing with statement: ‘Able to discuss any problem’	76% of the patients seen by a trainee vs. 91% of those seen by faculty (*p* < 0.02) found that they could discuss any problem
	Effect of training level (trainee vs. faculty) on patient mean satisfaction with *given explanation* of child’s condition and treatment	No statistically significant difference M = 4.9 (SD nr) with effect of training level coefficient 0.02 (*p* = 0.13)
* Monk et al., 2006*	Effect of training level (trainee vs. faculty) on patient mean satisfaction with *given instruction*	No statistically significant difference M = 4.8 9 (SD nr) with effect of training level coefficient 0.06 (*p* = 0.07)
* Sheets et al., 1991*	Mean satisfaction score of patients seen by trainees vs. seen by faculty for *given explanation*	No statistically significant difference M (SD nr) trainees 1.1 vs. faculty 1.2 (*p* > 0.05)
	Mean satisfaction score of patients seen by trainees vs. seen by faculty on *understanding of given explanation*	No statistically significant difference M (SD nr) trainees 1.2 vs. faculty 1.1 (*p* > 0.05)
* Li et al., 2018*	*Listening*: Percentage agreeing with: “Provider listened carefully to you,” of patients seen with trainee involvement vs. those seen without trainee involvement	91.1% (*n* = 8664) agreement with trainee involvement vs. 93.5% (*n* = 9610) without trainee involvement (*p* < 0.001)
	*Explanation*: (1) Percentage agreeing with: “Provider gave easy-to-understand instructions,” of patients seen with trainee involvement vs. those seen without trainee involvement (2) Percentage agreeing with: “Provider explained in a way you understood,” of patients seen with trainee involvement vs. those seen without trainee involvement	(1) 89.9% (*n* = 8738) agreement with trainee involvement vs. 92.2% (9532) without trainee involvement (*p* < 0.001) (2) 90.7% (*n* = 8621) agreement with trainee involvement vs. 93.3% (9578)without trainee involvement (*p* < 0.001)
	*Explanation*: Agreement with: “My doctor explains the reasons for procedures and test,” of patients seen by a trainee vs. those seen by faculty	UC: Patients seen by faculty were more likely to score high on explanation skills compared to patients seen by a trainee OR 3.2 (95% CI:1.3–7.8) VA clinic: No statistically significant difference OR 1.9 (95% CI:0.8–5.4)
	Satisfaction with *explanation* of what was done for you of patients seen by a trainee vs. patients seen by faculty	Patients seen at the UC are more likely to be satisfied when seen by faculty than those seed y trainees OR 2.6 (95% CI: 1.1–6.6) VA clinic: No statistically significant difference OR 1.8 (95% CI: 0.8–4.2)
* Yancy et al., 2001*(split on site: university clinic (UC) or veteran affairs (VA) clinic)	*Listening*: Agreement with: “My doctor listens very carefully,” of patients seen by a trainee vs. those seen by faculty	UC: Patients seen by faculty were more likely to score high on listening skills compared to patients seen by a trainee OR 4.1 (95% CI:1.6–10.4) VA clinic: No statistically significant difference OR 2.6 (95% CI:0.9–6.9)
	*Abilities*: Agreement with: “I have some doubts about the ability of my doctor,” of patients seen by a trainee vs. those seen by faculty	UC: Patients seen by faculty were more likely to score low compared to patients seen by a trainee OR 2.6 (95% CI:1.1–6.1) VA clinic: No statistically significant difference OR 1.6 (95% CI:0.9–2.8)
	*Attention*: Agreement with: “I feel there are some issues my doctor has not given enough attention,” of patients seen by a trainee vs. those seen by faculty	UC: No statistically significant difference OR 2.4 (95% CI: 1.0-5.7) VA clinic: No statistically significant difference OR 1.7 (95% CI: 0.9–3.2)
* Nakar*, et al.,* 2010*	Percentage agreeing with statement: “The trainee *explains* my problem as well as the regular doctor” (*n* = 304)	83.4% agreement
* Griffith et al., 2023*	Mean satisfaction score of patients seen by dermatologist or trainees for *professional explanations* of problem/condition	No significant difference M (SR nr): faculty 4.9 vs. trainee 4.8 (95%CI: 0.1–0.1) (scale 1–5)
	Mean satisfaction score of patients seen by dermatologist or trainees for *professional discussion* of treatments	No significant difference M (SR nr) : faculty 4.9 vs. trainee 4.8 (95%CI: 0.1–0.2) (scale 1–5)
* Rodrigues et al., 2023*	Percentage that answered completely on the question: ‘Was the problem you considered most important *discussed*?’ (*n* = 1387)	No significant difference Faculty 83% vs. trainee 81%
	Percentage that answered completely on the question: ‘Did your provider *listen* carefully to what you had to say?’ (*n* = 1387)	No significant difference Faculty 89% vs. trainee 88%
	Percentage that answered completely on the question: ‘Did our provider *explain* your problem or health status to you?’ (*n* = 1375)	Patients are more satisfied with the explanation given by faculty Faculty 76% vs. trainee 67% (*p* = 0.01)
	Percentage that answered completely on the question: ‘Did your provider *explore* how manageable recommended treatment or advice would be for you?’ (*n* = 1320)	Patients are more satisfied with the exploration done by faculty Faculty 78% vs. trainee 72% (*p* = 0.03)
	Percentage that answered completely on the question: ‘Were you able to *discuss* all your questions or worries?’ (*n* = 379)	Patients are more satisfied with the discussion of faculty Faculty 70% vs. trainee 64% (*p* = 0.047)
* Sherbuk et al., 2019*	Response as always to the question ‘did your primary doctor explain things in a way that was easy to understand?’ (*n* = 75)	No statistically significant difference 77% (HIV-specialist) vs. 88% (trainee)
	Response as always to the question ‘did your primary doctor listen carefully?’ (*n* = 75)	No statistically significant difference 83% (HIV-specialist) vs. 100% (trainee)
* Jaturapatporn et al., 2007*	Patients mean assessment score on doctor’s communication skill compered between faculty, trainees and general doctors	Patients of faculty and trainees are more satisfied than those seen by general doctors M (SD): faculty 69.8 (17.5)/trainee 69.8 (14.2)/general doctors 65.1 (14.2) (*p* < 0.001)
Trainee vs. faculty – Satisfaction with doctors’ behavior		
* Bradley et al., 1981*	Percentage of patients seen by a trainee (*n* = 42) vs. those seen by faculty (*n* = 206) that found atmosphere during consultation *not* relaxed	48% of patients seen by trainee vs. 10% of those seen by faculty found the atmosphere not relaxed (*p* < 0.001)
* Nakar et al., 2010*	Percentage agreeing with statement: “Treatment from the trainees is as *professional* as the regular doctor” (*n* = 304)	78.9% agreed that treatment by the trainee is as professional as the regular doctor
* Thornett et al., 2001*	Mean satisfaction score with *professional care* from patients seen by a trainee vs. patients seen by GPs	No statistically significant difference M (SD); trainees 79.6 (SD13.6) vs. GPs 79.3(10.5) (*p* = 0.91)
* Monk et al., 2006*	Effect of training status (trainees vs. faculty) on caregiver (parent) satisfaction with a physician’s *caring attitude*	Caregivers seen by trainees were less satisfied compared to those seen by faculty M (SD nr) 4.9, effect training level coefficient = 0.05 (*p* = 0.006)
* Rodney et al., 1986*	Patients mean satisfaction score with *humanenes*s of those seen by a trainee vs. those seen by faculty	No statistically significant differences M (SD nr): faculty 79.5 vs. trainee 69.7 (*p* = 0.2)
* Sheets et al., 1991*	Mean patient satisfaction score for *compassion* for patients seen by a trainee vs. patients seen by faculty	Patients seen by faculty scored higher on compassion for patients than those seen by trainees M (SD nr): trainees 1.1 vs. faculty 1.2 (*p* < 0.01)
* Yancy et al., 2001* (split on site: university clinic (UC) or veteran affairs (VA) clinic)	*Respec*t: Agreement with: “My doctor always treats me with the highest respect,” of patients seen by a trainee vs. those seen by faculty	UC: Patients seen by faculty were more likely to agree OR 7.5 (95% CI: 2.5–22.6) VA clinic: Patients seen by faculty were more likely to agree OR 5.1 (95% CI: 2.6–10.0)
	*Kindnes*s: Agreement with: “My doctor could have been kinder and more considerate of my feelings,” of patients seen by a trainee vs. those seen by faculty	UC: No statistically significant difference OR 1.9 (95% CI: 0.9–3.9) VA clinic: No statistically significant difference OR 1.6 (95% CI: 0.9–2.8)
	*Personal manner*: Satisfaction with personal manner (courtesy, respect, friendliness) of patients seen by a trainee vs. patients seen by faculty	No statistically difference on both sides UC: 3.2 (95% CI: 1.2–8.5) VA clinic: 2.4 (95% CI: 1.1–5.3)
* Li et al., 2018*	Percentage that is satisfied with showed respect of patients seen by a trainee and those who did not see a trainee	Patients seen by a trainee are less satisfied 93% (*n* = 9476) vs. 95% (*n* = 10266) (*p* < 0.001)
* Sherbuk e al., 2019*	Response “Always” on the question “”did your primary doctor show respect for what you had to say?” (*n* = 75)	No statistically significant difference 85% (HIV-specialist) vs. 96% (trainee); not significant

#### Trainee vs. faculty: Overall satisfaction (*n* = 8) and perceived quality of care (*n* = 3)

In six [19, 21, 22, 28, 39, 40] out of eight [19, 21, 22, 28, 29, 35, 39, 40] studies, no difference in overall satisfaction was found between patients seen by a trainee and those seen by a specialist. In two studies, patients seen by faculty were more satisfied [29, 35]. Perceived quality of care was measured in three studies [20, 30, 33]. Rodney et al. [33]. did not find a significant difference between faculty (GPs) and trainees. Allen et al. [20] reported that 61.3% of surveyed patients reported no difference in the quality of care between trainees and faculty, but 18.2% did not agree. Blanchard et al. [30] reported that 40% of patients were not significantly different.

#### Trainee vs. faculty: Satisfaction with communication skills (*n* = 11)

Studies regarding communication skills report patients’ satisfaction with doctors’ explanation skills [25, 27, 28, 32, 34, 35, 40, 41], listening skills [28, 35, 41], attention-related skills [35], instruction [27], discussion [28, 31, 43] and overall ability [11, 35, 39]. Most studies did not find a significant difference between trainees and faculty, with the exception of four studies [28, 31, 35, 41]. Among these, three reported lower satisfaction with trainees than with faculty in terms of explanation skills [28, 35, 41], two studies noted lower satisfaction with listening skills [35, 41], and two indicated lower satisfaction with discussion skills [28, 31] when comparing trainees to faculty.

#### Trainee vs. faculty: Satisfaction with doctor behavior (*n* = 9)

Nine studies reported on the following aspects of trainee behavior: professionalism [29, 32], caring attitudes [27], humanness [33], compassion [34], kindness [35], atmosphere [31] and respect [35, 40, 41]. Patients were significantly more satisfied with faculty than with trainees in terms of caring attitudes [27], respect [35, 41], compassion [34] and a relaxed atmosphere [31].

### Willingness

Eightteen studies [10, 12, 13, 16–18, 20, 23, 26, 30, 32, 36, 42, 44–48] reported patients’ willingness to involve a trainee in their care. The acceptance range varied between 38.7% [26] and 78.3% [18]. In two studies [26, 44], patients’ willingness depended on the level of supervision [26] or the complaint at hand [44]. Four studies explored patients’ reasons for accepting trainees [13, 18, 45, 46], with the patient’s wish to contribute to the learning of future doctors being the main reported reason. Three studies reported that the main reasons for patients refusing a trainee were ‘the wish to stay with their own docter’, ‘repeating history’ or ‘a previous bad experience’ [13, 18, 45]. Table 2 gives an overview.

**Table 2:** Patient overall willingness and reasons for refusal or acceptance

Study	Outcomes	Results
Overall willingness		
* Allen et al., 1981*	Percentage agreement with not minding having a trainee present during a consultation (*n* = 258)	72.1% agreed no one would mind having a GP trainee present during a consultation; 19.0% disagreed
* Bain et al., 1995*	Agreement with having no objection against a trainee sitting in on a consultation (*n* = 266)	54% would have no objection to a trainee sitting in on a consultation; for 35% this depended on the reason for consultation; 4% objected
	Agreement with having no objection against a consultation with a trainee alone (*n* = 266)	33% would have no objection to consulting a trainee alone; 28% would not object but preferred their own GP; for 27% it depended on the complaint; 7% would object
* Blanchard et al., 1977*	Percentage of refusal of trainee involvement (*n* = 285)	7.6% would refuse a trainee if given a choice
* Chambers et al., 2022*	Percentage of patients who would like to have a resident assist in care (*n* = 194)	68% yes
* Haider et al., 2009*	Percentage of patients accepting trainee participation (*n* = 115)	78.3% felt comfortable and accepted trainee participation; 21.7% refused participation
* Mantica et al., 2022*	Percentage of willing to attend resident for outpatient visit (*n* = 2587)	6.0% not willing, 6.1% doesn’t know, 26.2% willing if supervised, 23% willing if expert resident and 38.7% willing
* Reichgott et al., 1983*	Percentage of patients accepting a trainee in their care (*n* = 195)	73% willing to allow a trainee to be involved in their care
* Malcolm et al., 2008*	Percentage of patients who would choose to have trainee involved in their care (*n* = 251)	71.2% would choose to have a trainee involved; 23.6% would not; 5.2% is unsure
* Heathcote et al., 2007 & Heathcote et al., 2008*	Number of patients who would be willing to see a trainee	97 patients would be willing to see a trainee at least sometimes or were undecided
Reasons for acceptance		
* Chambers et al., 2022*	Reasons why patients chose to see residents (*n* = 194)	79,5% Help train future surgeons, 16.7% confidence in this office; 12.9% have had residents involved in care before and it was a good experience; 22.7% enjoy a teaching environment; 6.1% shorter wait time; 0.75% extra set of eyes; 0.75% brother is resident.
* Haider et al., 2009*	Patient reasons to allow a trainee to be involved in their care (*n* = 115)	81.1% trainees have to learn; 15.6% trainees are future consultants
* Heathcote et al., 2007 & Heathcote et al., 2008*	Patient reasons to allow a trainee in their care (*n* = 103)	49% felt obliged to visit a trainee; 84% wanted to help the trainee doctor; 59% perceived personal benefits when visiting a trainee
* Malcolm et al., 2008*	Top three reasons to allow the involvement of a trainee (*n* = 170)	1. Contribute to the training of future doctors (61.8%) 2. To obtain a second opinion (20%) 3. Trainees are more up-to-date (11.2%)
Reasons for refusal		
* Chambers et al., 2022*	Reasons why patient chose not to see resident (*n* = 194)	13.2% less skills; 17% personal reason; 4% had a bad experience prior to this resident; 5.7% make my appointment longer; 52.8% only want my doctor involved in my care; 1.9% VA patient
* Haider et al., 2009*	Patient reasons to reject a trainee (*n* = 115)	Shyness and lack of privacy (7%); consultation would take longer (4.3%); previous bad experience (10.4%)
* Malcolm et al., 2008*	Patient top three reasons to refuse a trainee (*n* = 59)	1. Continue relationship with their own family doctor (54.2%) 2. Avoid need to repeat history (18.6%) 3. Residents are less experienced (16.9%)

### Influencing factors

Fourteen studies [10, 12, 16, 17, 20, 23, 26, 32, 36–38, 42, 47, 48] described various factors that might affect patient satisfaction or willingness. These factors can be categorized according to patient characteristics (age, educational level, sex, self-reported health status and previous visits to the trainee) and are related to patients’ reasons for consultation. Table 3 provides an overview of patient characteristics, and Table 4 provides an overview of the influence of the reason for consultation.

**Table 3:** Effects of patient characteristics on patient satisfaction and willingness

Effect of patient characteristics		
Study	Outcomes	Results
Satisfaction		
	Patient satisfaction with the *received treatment* from the trainee	*Age*: Older patients are more satisfied than younger patients OR 1.48 (95% CI: 1.18–1.91)
		*Gender*: No statistically significant difference OR 1.54 (95% CI: 0.72–3.45)
		*Education*: Patients with a higher education are more satisfied than those with a lower education OR 2.77 (95% CI:1.30–6.29)
* Labgaa et al., 2014*	Patient satisfaction with the *skills* of trainee	*Age*: Older patients are more satisfied than younger patients OR 1.48 (95% CI: 1.18–1.82)
		*Gender*: No statistically significant difference OR 1.30 (95% CI: 0.65–2.63)
		*Education*: Patients with a higher education are more satisfied than those with a lower education OR 2.06 (95% CI: 1.06–4.16)
	Patient satisfaction with *behavior* of trainee	*Age*: Older patients are more satisfied than younger patients OR 1.35 (95% CI: 1.10–1.68)
		*Gender*: No statistically significant difference OR 1.56 (95% CI: 0.77–3.25)
		*Education*: No statistically significant difference OR 1.42 (95% CI: 0.73–2.84)
		*Gender*: Male patients are more satisfied than female patients OR 2.73 (95% CI: 1.45–5.10)
* Nakar et al., 2010*	Patient satisfaction with received treatment from a trainee (“Residents are as professional as the regular doctor”)	*Previous visits to the trainee*: Patients who visited the trainee before are more satisfied than those who did not OR 1.14 (95% CI: 1.01–1.28)
		*Frequency of visits to the clinic*: Frequent visitors are less satisfied than patients who visit the clinic less frequently OR 0.91 (95% CI: 0.85–0.98)
		*Age, Education, Employment Status, Marital Status, Self-rated Health status*: No statistically significant univariate relationships with outcome measure.
		*Expected approval regular GP*: Patients who think that their regular GP approves the visit to the trainee are more satisfied Response* = 5 (5-point scale)*: OR 18.05 (95% CI: 3.84–84.76) Response *= 4 (5-point scale)*:OR 5.88 (95% CI: 1.34–25.88)
		*Length of time with current GP*: Patients with a regular GP whom they are seeing less than one year are more satisfied with trainees than patients without regular GP OR 2.24 (95% CI: 1.31–3.85) Difference between patient who have a regular GP for 1–4 years vs. 5–10 years of longer than 10 years was not significantly different
* Bonney et al., 2012*	Patient satisfaction with care received from trainees	*Patient age*: Older patients tend to be more satisfied OR 1.03 (95% CI: 1.00-1.06)
		*Gender*: Male patients are more satisfied OR 0.73 (95% CI : 0.57–0.94)
		*Self-rated health No statistically significant effect*
		*Chronic condition No statistically significant effect*
		*Interpersonal trust (IPT)*: Patients with high IPT scores (suggested to be related to valuing continuity of care) are less satisfied OR 0.48 (95% CI: 0.32–0.74)
		*System trust (ST)*: Patients with high ST scores are more satisfied OR 6.31 (95% CI: 4.30–9.26)
	Patient satisfaction with care received from trainees related to scores on interpersonal trust (IPT), system trust (ST) and interpersonal continuity factors (IPC)	*Interpersonal continuity (IPC): No significant relationship*
		*Interpersonal trust (IPT)*: Patients with high IPT are less satisfied OR: 0.48 (95% CI: 0.32–0.74)
* Bonney et al., 2014*	Predictors of high scores on IPT, ST and IPC	ST: Age over 75 years and high self-rated health predict high scores on System Trust Age > 75 years (OR 1.57; 95% CI: 1.17–2.10) High self-rated health (OR 1.68; 95% CI: 1.24–2.27)
		IPC: Being a female, having a chronic illness and/or being patient at the same practice for over 5 years predict high scores on Interpersonal continuity.Female gender (OR 2.37; 95% CI: 1.60–3.52),Having a chronic illness (OR 2.38; 95% CI: 1.29–4.39),Being at the same practice > 5 years (OR 4.44; 95% CI: 2.71–7.28)
		IPT: Low self-rated health and female gender predict high scores on Interpersonal TrustLow self-rated health (OR 1.46; 95% CI: 1.05–2.03)Female gender (OR 1.85; 95% CI: 1.34–2.57)
* Crawford et al., 2005*	Patient satisfaction with care received from trainees	*Insurance type*: No significant association
		*Education level patients*: No significant association.
Willingness		
* Bonney et al., 2012* [12]	Frequency of visits to the trainee	*Expected approval regular GP*: Patients who think that their regular GP approves the visit to the trainee, visit the trainee more often *Response = 5 (5-point scale)*: OR 4.33 (95% CI:1.69–11.09) Response *= 4 (5-point scale)*: OR 3.16 (95% CI: 1.23–8.12)
		*Length of time with current GP*: Patients who have a regular GP, visit the trainee less frequently.Length of time 1–4 years: OR 0.42 (95% CI: 0.26–0.68)Length of time < 1 year: OR 0.34 (95% CI: 0.17–0.67)
* Bonney et al., 2014*	Frequency of visits to the trainee related to scores on interpersonal trust (IPT), system trust (ST) and interpersonal continuity factors (IPC)	*IPC*: Patient with high scores on IPC visit the trainee less frequentlyOR 0.42 (95% CI: 0.24–0.74)
		*IPT: No significant relationship*
		*ST*: Patients with high ST score visit the trainee more frequentlyOR 1.99 (95% CI: 1.53–2.60)
	Predictors of high scores on interpersonal trust (IPT), system trust (ST) and interpersonal continuity factors (IPC)	*See above in Satisfaction*
		*Sex*: No statistically significant effect
		*Income: *No statistically significant effect
		*Race:* No statistically significant effect
* Reichgott et al., 1983*	Patient willingness to allow a trainee to be involved in their care	*Education level:* Patients with a lower education level were more willing than patient with a higher education level (*p* = 0.006)
		*Insurance:* Patients who were insured by medicare were more willing to accept trainees in their care *(p < 0.0001)*
		*Previous visits to the resident:* Patients who had previous experience with a trainee and were satisfied were more willing to allow a trainee* (p < 0.001)*
		*Age*: No statistically significant effect
		*Age (40 +* vs. *< 30 years*): No statistically significant effect OR 1.5 (95% CI: 0.8–2.5)
	Patient allows a trainee to perform a psychological examination	*Gender (male* vs. *female*): No statistically significant effect OR 1.0 (95% CI: 0.7–1.5)
		*Education level* (low vs. high): No statistically significant effect OR 1.3 (95% CI: 0.9-2.0)
		*Previous visits to the clinic* (0–2 vs. 6+): No statistically significant effect OR 0.7 (95% CI: 0.4–1.1)
		*Occupation and household income: no significant effect*
* AlGhamdi et al., 2014 *[36]	Patient acceptance of a trainee to perform a skin biopsy	*Age* (40 + vs. < 30 years): No statistically significant effect OR 1.2 (95% CI: 0.6–2.1)
		*Gender concordance* Female patients disagree skin biopsy more often when performed by a male resident OR 2.0 (95% CI: 1.1–3.6)
		*Education level* (low vs. high): No statistically significant effect OR 1.0 (95% CI: 0.7–1.6)
		*Previous visits to the clinic* (0–2 vs. 6+): No statistically significant effect OR 0.7 (95% CI: 0.4–1.2)
		*Occupation and household income*: no significant effect
		Breathing problems:> 40 years: 3% prefer trainee, 56% prefer usual doctor, 36% no preference < 40 years: 4% prefer trainee, 43% prefer usual doctor, 49 % no preference *P* < 0.005
	*Age*: Patient preference on which doctor to visit (trainees vs. usual doctor vs. no preference), according to various clinical scenarios, split on patient under or over 40 years	High blood pressure:> 40 years: 7% prefer trainee, 55% prefer usual doctor, 29% no preference < 40 years: 7% prefer trainee, 44% prefer usual doctor, 44% no preference *P* < 0.0005
		Sick child with high temperature:> 40 years: 6% prefer trainee, 38% prefer usual doctor, 42% no preference < 40 years: 6% prefer trainee, 41% prefer usual doctor, 49% no preference No significant difference
		Relationship problems: > 40 years: 8% prefer trainee, 63% prefer usual doctor, 20% no preference < 40 years: 15% prefer trainee, 51% prefer usual doctor, 28% no preference *P* < 0.0005
* Murphy et al., 1995*		Sore throat: > 40 years: 9% prefer trainee, 24% prefer usual doctor, 59% no preference < 40 years: 12% prefer trainee, 16% prefer usual doctor, 69% no preference *P* < 0.0005
		Breathing problems: + visit: 4% prefer trainee, 45% prefer usual doctor, 46% no preference - visit: 4% prefer trainee, 50% prefer usual doctor, 40% no preference No significant difference
		High blood pressure: + visit: 7% prefer trainee, 43% prefer usual doctor, 44% no preference - visit: 7% prefer trainee, 57% prefer usual doctor, 30% no preference *P* < 0.0005
	*Previous visits to trainee: Patient preference on which doctor to visit (trainee vs. usual doctor vs. no preference), according to various clinical scenarios, split on patients who previously visited the trainee (+) and those who did not (-)*	Sick child with high temperature: + visit: 6% prefer trainee, 38% prefer usual doctor, 48% no preference - visit: 4% prefer trainee, 43% prefer usual doctor, 42% no preference *P* < 0.05
		Relationship problems: + visit: 13% prefer trainee, 52% prefer usual doctor, 27% no preference - visit: 10% prefer trainee, 62% prefer usual doctor, 20% no preference *P* < 0.0005
		Sore throat: + visit: 11% prefer trainee, 13% prefer usual doctor, 70% no preference- visit: 10% prefer trainee, 30% prefer usual doctor, 54% no preference*P* < 0.0005
* Mantica et al., 2022*	Willingness to attend outpatient visit with resident	*Gender*: Female patient are less willingOR 0.51 95% (CI 0.36–0.73)
		*Previous visit*: Patients who come for a follow-up visits are less willingOR 0.69 (95%CI 0.36–0.73)
		*Age*: Patients who are older are more willingOR 1.02 I95%CI 1.00-1.03)
		*Disease*: Having oncological disease more willing OR 1.53 (95%CI 1.04–2.27)
		*Being retired and education level*: no significant effect
* Rifkin et al., 2002*	Percentage of female patients willing to allow a male trainee to perform a pelvic examination	*Age*: No statistically significant effect (< 25 years vs. 26–40 year vs. > 40 years), *p* = 0.07
		*Race: No statistically significant effect (Non-Caucasian vs. Caucasian), p = 0.59*
		*Income: No statistically significant effect (< 20k vs. 20-40k vs. > 40k dollar/year) p = 0.12*
		*Education*: < 12 years; 12–15 years > 16 years, *p* = 0.12
* De Bever et al., 2022*	Willingness to visit a trainee	Patients who visited a trainee, and where happy with the visit, tend to form a bond with the trainee. This bond is important to form a trusting relationship between patient and doctor, and is of influence on patients’ willingness to consult a trainee.
* Carruthers et al., 2015*	Agreement with statement ‘If a resident were to be involved in my care, I would prefer that they be further along in their program’	*Level of training*: M = 3.6 (SD: 1) for reconstructive surgery patients and M = 3.3 (SD 1.1) for cosmetic surgery patients. No statistically significant difference between groups
	Agreement with statement ‘If a resident were to be involved in my care, I would feel more comfortable if they were of the same sex as me’	*Gender*: M: 2.7 (SD: 1.0) for reconstructive surgery patients and M: 2.8 (SD:1.2) for cosmetic surgery patients. No statistically significant difference between groups

**Table 4:** Effects of patients’ reasons for consultation on patients’ willingness to allow trainee involvement in their care

Effect of patient’s reason for consultation		
Study	Outcomes	Results
*Allen et al., 1981*	Percentage agreement with statement: “It does not matter which doctor is seen for an urgent problem”	75.2% agree; 19.8% disagree (n = nr*)
	Percentage agreement with statement: “In long-standing illness, one does not want to see a trainee”	47.7% agree; 36.4% disagree (n = nr)
*Bonney et al., 2009*	Patient reasons for acceptance of trainee involvement, split on having an urgent vs. long-standing illness	Long-standing illness: Preference for own doctor because of continuity of care, trust this doctor. Urgent complaints: Patients have no relationship with trainees, although for urgent matters or convenience they are willing to consult the trainee
		Breathing problems: 4% prefer trainees, 46% prefer usual doctor, 44% no preference
		High blood pressure: 6% prefer trainees, 48% prefer usual doctor, 37% no preference
*Murphy et al., 1995*	Patient preference on which doctor to visit (trainees vs. usual doctor vs. no preference), according to various clinical scenarios (*n* = 1510)	Sick child with high temperature: 5% prefer trainees, 39% prefer usual doctor, 45% no preference
		Relationship problems: 12% prefer trainees, 55% prefer usual doctor, 24% no preference
		Sore throat: 10% prefer trainees, 20% prefer usual doctor, 63% no preference
*Carruthers et al., 2015*	Patient comfort to allow a trainee to observe a consultation for reconstructive surgery vs. cosmetic surgery	Patients seen for reconstructive surgery were more willing to allow a trainee to observe a consultation than patients seen for cosmetic surgery Mean willingness reconstructive 4.1 (SD 0.93) (scale 0–5) vs. mean willingness cosmetic 3.6 (SD 1.09); *p* < 0.01.
*Crawford et al., 2005(37)*	Willingness to allow a trainee to be involved in their care according to various procedures (*n* = 191)	93.3% acceptance for history taking; 87.2% for psychological examination; 44.7% for prescribing medications; 43.6% for skin biopsy; 19.7% for surgical excision of skin cancer; 55.3% for cosmetic counselling; 74.5% for preventive counselling
*Reichgott et al., 1983*	Percentage of patients willing to allow a trainee to be involved in their care according to various procedures (*n* = 143)	93% history taking; 78% psychological examination; 41% order and interpret tests; 30% prescribe treatment
*Nakar et al., 2010*	Percentage of patients who agreed with: “There are specific test I prefer the regular doctor to perform” (*n* = 304)	42.1% agreed; 79,1% preferred the senior doctor for examination of intimate areas; 17% for blood test
	Percentage of patients who agreed with: ‘There are topics which I feel uncomfortable discussing with the resident’ (*n* = 304)	30.3% agreed
*AlGhamdi et al., 2014*	Percentage of patients willing to allow a trainee to be involved in their care according to various procedures (*n* = 742)	87.6% acceptance for history taking; 86.3% acceptance for providing advice; 37.6% acceptance for skin biopsy; and 29.5% acceptance for malignant skin tumor excision
*de Bever et al., 2022 *[16]	Factors that influence patients’ willingness to consult a GP trainee (qualitative)	Patients’ willingness to visit a trainee is fluid, and depend on the extent a trainee can fulfil patients’ preferences. Patient preferences strongly depend on their reason for consultation. For small or urgent complaints they have no preference in doctor, while for long-standing illness or (potential) risky diseases they prefer a doctor with whom they have a trusted relationship

*nr not reported

### Patient characteristics

#### Age (*n* = 9)

Three studies suggested that older patients are more satisfied with trainees than younger patients are [12, 17, 38]. Bonney et al. [17]. reported that patients over 75 years (with high trust in their GP or the GP practice) were more satisfied with trainees. Nakar et al., did not find any influence of age on patient satisfaction [32].

Six studies described the relationship between age and willingness to see a trainee [17, 26, 36, 42, 47, 48]. Bonney described greater system trust in patients over 75 years of age, but there was no relationship between age and other parameters [17]. Murphy reported that for most medical problems, older patients prefer their usual doctor [47], whereas Mantica reported that older patients are more willing to see a trainee [26]; other studies have reported no difference in willingness with respect to age [36, 42, 48].

#### Gender (*n* = 8)

Among the four studies reporting on the relationship between genderand satisfaction [12, 17, 32, 38], three reported that male patients were more satisfied with trainees than female patients were [12, 17, 32], and one reported no difference [38]. Five studies described the relationship between gender and the willingness to see a trainee [17, 23, 26, 36, 42]. In two studies, females were less willing to consult a trainee [17, 26]. According to Bonney et al., this is related to females with low self-rated health and who place high value on interpersonal continuity [17]. AlGhamdi et al. reported that female patients more often refuse male residents when a skin biopsy is performed than when a physical examination is not performed [36].

#### Educational level (*n* = 6)

Three studies described the relationship between educational level and satisfaction [32, 37, 38]. Labgaa et al. reported a positive relationship between educational level and trainee satisfaction with treatment and skills but no effect on satisfaction with behavior [38]. The others find no relation [32, 37]. With respect to willingness, only one study reported greater willingness among patients with a lower educational level [42], whereas other studies reported no relation [26, 36, 48].

#### Previous visits to the trainee (*n* = 5)

Nakar et al. reported that patients who visited a trainee before the trainee was more satisfied [32]. This was confirmed in studies on willingness, which all revealed a positive correlation between previous visits and the willingness to see the trainee [16, 42, 47]. Faugeax et al., reported that patients who had good experiences visiting trainees were also more satisfied with trainees’ communication skills [24]. None of the included studies specified whether continuity referred to the same trainee or any trainee.

#### Self-reported health (*n* = 3)

Three studies reported health status and satisfaction or willingness [12, 17, 32]. Two studies reported no relationship between patients’ satisfaction and their self-reported health [12, 32]. Bonney et al., reported that patients with low self-reported health are less willing to see a trainee, usually because they place high value on interpersonal continuity [17]. However, patients with high self-rated health who have high levels of trust in the practice or in a GP (system trust) are willing to see the trainee [17].

### Reasons for consultation

Studies have shown that most patients are willing to consult a trainee for urgent complaints but not for long-standing illnesses [10, 20, 47]. Moreover, a decreasing number of patients are willing to allow a trainee to perform a task with increasing difficulty [32, 36, 37, 42]. Bonney et al., and de Bever et al., further emphasized that patients tend to prefer their own doctor for long-standing illnesses and complex diseases, while they express no preference for urgent or minor ailments [10, 16]. Whether they are willing to consult the trainee depends on the extent to which a trainee can fulfil patients’ preferences concerning their doctor, such as availability or gender [16]. No studies reported any effect on satisfaction with patients’ reasons for consultation.

## Discussion

This review aims to describe patient perspectives—in terms of satisfaction and willingness—on trainee involvement in their care and identify the factors influencing these perspectives to optimize patient participation in the learning of trainees in clinical practice. Thirty-eight studies were included; outcomes were defined in various ways, showing considerable variation in specificity. To bring structure to this diversity, we used *patient satisfaction* and *willingness* as overarching themes.

Twenty-one studies reported patient satisfaction with the trainee. Most studies reported notably high satisfaction rates, often exceeding 90%. However, these rates were significantly lower in half of the studies than in the faculty assessments of communication skills and interpersonal competencies, such as caring attitudes and respect.

This is confirmed by our other theme: their willingness to allow a trainee in their care. Patients’ main reason for agreeing to see a trainee was to contribute to the training of new doctors, whereas the most frequently reported reason for refusing a trainee was the preference for continuity in their care, such as not wanting to repeat their medical history or wishing to remain with their regular doctor. The percentage of patients willing to involve a trainee ranged from 54% to 74%, which is lower than the reported satisfaction rates. This suggests that while patients may express satisfaction after seeing a trainee, they may be less inclined to involve them in ongoing care. Vaughn et al., identified similar findings in their review of patient perspectives on medical student involvement [8]. These results raise the question of whether satisfaction alone is an accurate indicator of patients’ views on learner involvement.

In addition to describing satisfaction and willingness, we also identified several factors that influence these views. The most prominent factors include low self-rated health, female sex, previous visits and the patient’s reason for consultation. Patients are generally more willing to involve a trainee in simple tasks (e.g., 97% are willing to have a trainee take their medical history), but willingness decreases considerably as the complexity of the task increases (e.g., only 16% are willing to have a trainee perform a skin cancer biopsy). Additionally, female patients and those with lower self-rated health are less likely to accept a trainee’s involvement. However, patients who have prior experience with trainees, whether through previous encounters with any trainee or through the development of a relationship with a specific trainee, tend to be more willing to engage and report higher satisfaction.

Drevs et al., suggested that patients make an appraisal of potential consequences before deciding whether to include a learner in their care [49]. Anxiety and perceived personal costs are the strongest predictors of whether patients allow a learner to participate. For more complex tasks, such as a skin cancer biopsy, patients perceive higher costs, including the risk of an unfavourable outcome, which may lead them to exclude trainees. For patients with long-standing illnesses, or those with low self-rated health, the perceived cost is often the loss of continuity of care, which is typically best provided by a trusted doctor. Trainees are not usually viewed as fulfilling the role of a trusted physician, although they may assume this role if a patient consults them multiple times [16]. This aligns with our finding that prior experience with a trainee positively influences patients’ perspectives on trainee involvement. This positive influence is likely related to increased familiarity with the trainee and the development of a bond between the patient and the trainee [4].

Despite these findings, it is difficult to draw strong conclusions from this review because of the low quality of the included studies. The mean Medical Education Research Study Quality Instrument (MERSQI) score was 8.5 out of 18 points, with a notable lack of evidence regarding the validity of the instruments used. Among the 38 studies included, only eight employed prevalidated questionnaires, and seven did not report any validity data.

### Limitations

All of the included studies reported highly positive results, with no studies explicitly indicating negative patient feedback about trainees. This could reflect bias, as patients may feel vulnerable or dependent when completing satisfaction questionnaires [50]. However, because satisfaction measures are not used for feedback purposes, we expect the bias to be minimal.

This review focused on ambulatory care settings, which have specific characteristics—such as the types and severity of diseases patients experience, greater autonomy in choosing their healthcare provider, and long-term care relationships—that make the findings context-specific and not directly transferable to inpatient hospital settings. Vaughn et al., previously investigated patient perspectives on medical students in inpatient settings and reported similar results [8]. It would be valuable to explore whether these findings also hold when the treating physician is a trainee.

An additional limitation is that most studies did not specify the degree of trainee involvement in patient care. Given that patients often express concerns regarding loss of continuity and increased personal costs, these concerns may be alleviated when the patient’s own physician remains directly involved in their care and supervises the trainee. Future research should consider the level of trainee participation to better understand its impact on patient perspectives.

### Implications for research and education

This review highlights the need for high-quality research to better understand patient attitudes and perspectives toward trainee involvement in clinical care. As most included studies reported on satisfaction and willingness, future research should prioritize qualitative approaches to explore these perspectives in greater depth. Such studies are essential to gain richer insights into patients’ experiences, motivations, and concerns, thereby underscoring the importance of understanding the patient perspective in shaping educational and clinical practices.

Our findings suggest that patients are generally satisfied after encountering trainees and are often willing to see them again, particularly when the reason for consultation is straightforward or when there has been prior contact with the trainee. However, patients reported lower satisfaction with trainees’ caring attitudes, respect, compassion, and ability to create a relaxing atmosphere than faculty did, highlighting the need to strengthen training in patient communication competencies. Moreover, the role of the attending physician is important in shaping patients’ acceptance of trainee involvement, as what the faculty communicates to patients about the trainee’s role and supervision influences their perceptions. This underscores the importance of faculty development, as faculty members must actively participate in fostering an educational environment that supports both patient-centered care and effective trainee learning.

Furthermore, providing continuity in trainee placement may enhance both patient trust and learning opportunities. This continuity should be actively facilitated by clinic scheduling practices that prioritize patient-trainee continuity.

## Conclusion

This review identified and summarized the findings of 38 studies on patient satisfaction and willingness concerning trainee involvement in their care and factors that might influence patient satisfaction and willingness in an ambulatory care setting. Although the results must be interpreted with care given the relatively low quality of many included studies, some tentative conclusions can be drawn. This review highlights a generally positive patient perspective on trainee involvement, with most patients expressing satisfaction with trainees and a willingness to be seen by them — particularly in cases involving straightforward consultations or when previous contact had occurred. However, patients reported lower satisfaction with trainees than with fully qualified physicians in domains related to caring attitudes, respect, compassion, and the ability to create a relaxing atmosphere. These findings underscore the importance of strengthening communication and interpersonal competencies in postgraduate medical training. In addition, continuity in trainee placement may enhance both patient trust and the educational value of clinical placements.

## Supplementary Information

Supplementary Material 1.

Supplementary Material 2.

Supplementary Material 3.

Supplementary Material 4.

Supplementary Material 5.

## Data Availability

Data is provided within the manuscript or supplementary information file.
