# Supplementary material for: Patient satisfaction and willingness as indicators for patient perspectives toward trainee involvement: a systematic review
Source: BMC Med Educ. 2025 Dec 22;25:1749. doi: 10.1186/s12909-025-08310-4 (PMC12751747; doi:10.1186/s12909-025-08310-4)
Supplement: Supplementary file 1 — Supplementary Material 1. [file 12909_2025_8310_MOESM1_ESM.docx]

**Appendix I – Search string per database**

MEDLINE (Ovid):

| # | Searches |
| --- | --- |
| 1 | Ambulatory Care/ or exp Ambulatory Care Facilities/ or exp Physicians/ or exp general practice/ or exp Primary Health Care/ or (physician* or doctor* or ambulatory care or ambulatory service* or outpatient*).ti,ab,kw. |
| 2 | "Internship and Residency"/ or (registrar* or residen* or trainee* or postgraduate* or post-graduate*).ti,ab,kw. |
| 3 | exp "Patient Acceptance of Health Care"/ or Physician-Patient Relations/ or exp Patient Satisfaction/ or (patient* adj9 (satisf* or attitude* or opinion* or trust* or feedback or experience* or happy or willingness or disadvantage*) adj12 (GPR* or registrar* or residen* or trainee* or postgraduate* or post-graduate*)).ti,ab,kw. |
| 4 | 1 and 2 and 3 |

EMBASE (Ovid):

| # | Searches |
| --- | --- |
| 1 | exp ambulatory care/ or outpatient department/ or exp physician/ or general practice/ or exp primary health care/ or (physician* or doctor* or ambulatory care or ambulatory service* or outpatient*).ti,ab,kw. |
| 2 | resident/ or (registrar* or residen* or trainee* or postgraduate* or post-graduate*).ti,ab,kw. |
| 3 | exp patient attitude/ or (patient* adj9 (satisf* or attitude* or opinion* or trust* or feedback or experience* or happy or willingness or disadvantage*) adj12 (GPRs or registrar* or residen* or trainee* or postgraduate* or post-graduate)).ti,ab,kw. |
| 4 | 1 and 2 and 3 |
| 5 | limit 4 to conference abstract status |
| 6 | 4 not 5 |

PsycINFO (Ovid):

| # | Searches |
| --- | --- |
| 1 | exp outpatient treatment/ or exp physicians/ or primary health care/ or (physician* or doctor* or ambulatory care or ambulatory service* or outpatient*).ti,ab,id. |
| 2 | medical residency/ or postgraduate training/ or (registrar* or residen* or trainee* or postgraduate* or post-graduate*).ti,ab,id. |
| 3 | exp client attitudes/ or (patient* adj9 (satisf* or attitude* or opinion* or trust* or feedback or experience* or happy or willingness or disadvantage*) adj12 (GPRs or registrar* or residen* or trainee* or postgraduate* or post-graduate*)).ti,ab,id. |
| 4 | 1 and 2 and 3 |

ERIC (Ovid):

| # | Searches |
| --- | --- |
| 1 | physicians/ or exp medicine/ or (physician* or doctor* or ambulatory care or ambulatory service* or outpatient care or consultant or faculty).ti,ab,id. |
| 2 | graduate medical education/ or (registrar* or residen* or trainee* or postgraduate* or post-graduate*).ti,ab,id. |
| 3 | attitudes/ or satisfaction/ or (patient* adj9 (satisf* or attitude* or opinion* or trust* or feedback or experience* or happy or willingness or disadvantage*) adj12 (GPRs or registrar* or residen* or trainee* or postgraduate* or post-graduate*)).ti,ab,id. |
| 4 | 1 and 2 and 3 |
