# Supplementary material for: Patient satisfaction and willingness as indicators for patient perspectives toward trainee involvement: a systematic review
Source: BMC Med Educ. 2025 Dec 22;25:1749. doi: 10.1186/s12909-025-08310-4 (PMC12751747; doi:10.1186/s12909-025-08310-4)
Supplement: Supplementary file 2 — Supplementary Material 2. [file 12909_2025_8310_MOESM2_ESM.docx]

**Appendix II – Study characteristics**

| **Study** | **Specialty & country** | **Setting** | **Study design** | **Aims** | **Participants** | **Sample size** | **Trainee level** | **Level of supervision trainee** |
| --- | --- | --- | --- | --- | --- | --- | --- | --- |
| **AlGhamdi et al., 2014** | Dermatology, Saudi-Arabia | 1 dermatology clinic at the King Khalid University Hospital in Riyad | Survey design | Evaluate patient perceptions, attitudes and satisfaction regarding resident participation | All patients visiting the clinic over two months seen by a resident | 900 eligible patients; 724 included (82%) | Not specified | Reactive supervision, double-checking after consultation |
| **Allen & Bahrami., 1981** | General practice, UK | 1 general practice | Survey design | To give patients the opportunity to express their attitudes toward trainees | All patients visiting the practice over a two-week period | 258 eligible patients | Not specified | Not specified |
| **Bain & Mackay., 1995** | General practice, UK | 3 general practices around Glasgow | Survey design | Examine patient views on their participation in training and assessment of trainees and qualified GPs | All patients visiting the practice over one day | 299 eligible patients; 266 included (89%) | Not specified | Not specified |
| **De Bever et al., 2022** | General practice, NL | 5 general practices around Amsterdam and Leiden | Qualitative interview study | Explore patients’ reasons for accepting or refusing trainees in their care, and which motives and ideas affect this decision. | Purposeful sampling & all patients visiting over one day | Not applicable | First and last year residents | Not specified |
| **Blanchard et al., 1977** | General practice, USA | 1 general practice in Mississippi | Survey design | Examining the effects on patient satisfaction after introducing residents | All patients visiting the clinic over four weeks, several months prior to introducing residents and all patients visiting the clinic five months after introducing residents | 870 eligible patients (388 1st period, 540 2^nd^ period); 560 included (275 1st period, 285 2nd period) (64%) | First year resident | Not specified |
| **Bonds et al., 2004** | Internal medicine, USA | 1 internal medicine primary care clinic at an academic hospital | Survey design | To determine the level of trust medically underserved patients have in their resident physicians | Patients were selected at random from the appointment schedule | 233 eligible patients; 217 included (93%) | Not specified | Not specified |
| **Bonney et al., 2009** | General practice, Australia | 3 general practices in New South Wales | Qualitative interview study | Exploring older patient attitudes toward residents | Patients above 60 years of age were invited to participate. Interested patients could contact the research team. | Not applicable | Not specified | Not specified |
| **Bonney et al., 2010** | General practice, Australia | 10 general practices in regional Australia (5 rural, 5 urban) | Survey design | Whether older patient acceptance of trainees would improve by strengthening the relational link between their regular GP around trainee consultations | Per GP practice, 50 sequential patients over 60 years of age who visited either the GP, the trainee or a nurse, were invited to participate | 500 eligible patients; 233 participated (47%) | Not specified | Not specified |
| **Bonney et al., 2012 & Bonney et al., 20 14** | General practice, Australia | 37 general practices across Australia (19 rural and 18 urban) | Survey design | Whether older patient acceptance of trainees would improve by strengthening the relational link between their regular GP around trainee consultations | Per GP practice, 50 sequential patients over 60 years of age who visited either the GP, the trainee or a nurse, were invited to participate | 1900 eligible patients; 911 participated (47.9%) | Not specified | Not specified |
| **Bradley et al., 1981** | General practice, UK | 1 health center in Exeter | Survey design, pre- and post-consultation | Exploring the expectation of patients, whether or not these expectations or met and the differences between trainees and GPs | All patients visiting the practice over one week. Antenatal patients were excluded. | 348 eligible patients; 248 participated (71%) | First-year resident | Not specified |
| **Brahmania et al., 2015** | Gastro-enterology, Canada | 1 GE clinic at the St Boniface Hospital, Winnipeg | Survey design | Comparison of patient satisfaction between residents and attending physicians | All patients visiting the clinic over one year. Patients seen for follow-up and with intellectual disabilities were excluded | 211 participants | First, second and third-year internal medicine residents. Fourth and fifth-year GI fellows | Reactive supervision: review with supervisor after consultation |
| **Chambers et al., 2022** | General surgery, USA | 1 general surgery office affiliated with North-East Georgia | Survey design | Understanding of the comfort level of patients having residents involved in their care, and if they knew what having a resident involved in their care meant | All patients visiting the office between 6 month | 657 eligible; 222 collected (34%) | Not specified | Not specified |
| **Crawford et al., 2005** | Dermatology, USA | Dermatology clinic of the University of Pennsylvania | Survey design | Assess patient attitudes toward resident participation | Patients scheduled to visit an attending physician over a 3-month period | 206 eligible patients; 191 participated (92.7%) | Not specified | Reactive supervision; double-checking after consultation |
| **Cao & Chen et al., 2018** | Dermatology, USA | A resident clinic (RC) and an urgent access clinic (UAC) | Survey design | Evaluation of patient satisfaction with resident involvement | All patients seen in the RC or UAC over two months | 127 participants (49 in RC and 78 in AUC) | Not specified | In RC the resident reactive supervision, in AUC under direct supervision |
| **Carruthers et al., 2015** | Plastic surgery, USA | 2 plastic surgery outpatient clinics in Ohio | Survey design | To determine if attitudes of patients toward resident participation differs between patients seen for aesthetic and for non-aesthetic issues | All first-time patients above 18 years in a 7-month time period | 119 participants | Not specified | Not specified |
| **Faugeroux et al., 2023** | Dermatology, France | Multiple dermatology centers in France | Survey design | Evaluate the perception of patients with atopic dermatitis or psoriasis regarding dermatology residents | All patients connected to PSO association or eczema association between October 2020 and February 2021 | 3154 included | Not specified | Not specified |
| **Griffith et al., 2023** | Dermatology, USA | 1 dermatology center at the University of Texas South Western | Survey design | Describing patient satisfaction with attending physician, physician assistant (PA) and residents | All patients who visited the department of dermatology between April 2019 and December 2021 | 12386 included | Third or fourth year trainees. | Not specified |
| **Haider et al., 2009** | Gynecology, Pakistan | Gynecology clinic of the Isra University Hospital | Survey design | Exploring patient views on trainee presence during consultation | Every third patient between 20-40 years of age visiting the clinic | 115 participants | First, second, third, fourth and fifth-year residents | Allowed to be present and observe the consultation |
| **Heathcote et al., 2007 & Heathcote et al, 2008** | Mostly general practice residents, UK | 1 family planning clinic in Cheshire | Survey design | To obtain patient views about the process of giving consent to resident involvement. To discover patient motivations about resident involvement | All patients visiting the clinic | 109 eligible patients; 103 participated (94.5%) | Not specified | Not specified |
| **Jaturapatporn & Dellow et al., 2007** | Family medicine, Thailand | 1 family medicine clinic at the Ramathibodi Hospital in Bangkok | Survey design | Comparison of patient satisfaction with faculty, residents and general doctors | Every fourth patient visiting the clinic over two weeks | 2500 eligible patients; 1870 participated (70%) | Not specified | Not specified |
| **Labgaa et al., 2014** | General practice, Switzerland | 1 academic walk-in center in Lausanne | Survey design | To assess patient satisfaction about the care they received from residents and their supervision by family doctors. The secondary aim was to describe walk-in patients’ demographic characteristics and to identify potential associations with satisfaction | All patients visiting the clinic over two weeks | 395 eligible patients; 184 participated (47%) | Not specified | Not specified |
| **Li et al., 2017** | Surgery, USA | One surgical outpatient clinic at San Diego Medical Center | Survey design | To assess whether resident participation in the outpatient setting had an impact on patient willingness to recommend the practice and overall patient satisfaction | All patients who visited the clinic in a 7-month time period | 73,834 eligible patients; 17,653 participated (23.9%) | Not specified | Not specified |
| **Malcolm et al., 2008** | General practice, Canada | 1 general practice affiliated with University of British Colombia, Vancouver | Survey design | Assessment of patient knowledge of resident training and role and their preferences regarding resident involvement | All patients visiting the practice over one month | 265 eligible patients; 251 participated (94.7%) | Not specified | Reactive supervision, case review after consultation |
| **Mantica et al., 2022** | Urology, Italy | 22 academic institutions over Italy | Survey design | To evaluate their [patients] perceptions of the quality of care provided by residents and to evaluate patients’ willingness to be managed by urology residents | All patients visiting an outpatient clinic as well patients visiting for diagnostic and/or surgical procedures, or upon discharge from the ward | 2587 participants | Not specified | Not specified |
| **Murphyet al., 1995** | General practice, Ireland | 10 general practices affiliated with the Dublin General Practice Vocational Training Scheme (4 urban, 3 rural, 3 mixed) | Survey design | Establishing patient attitudes toward trainees | All patients visiting a practice over two weeks | 2000 eligible patients; 1500 participated (75%) | Not specified | Not specified |
| **Monk et al., 2006** | Pediatrics, USA | 1 primary care, pediatrics hospital-based clinic in Ohio | Survey design | Comparison of caregiver satisfaction with residents and attending physician | All parents who visited the clinic over a 15-month period | 672 participants | First, second, third and fourth-year residents | Reactive supervision with double-checking after consultation for first year residents. Other residents, only paper review by the supervisor |
| **Nakar et al., 2010** | Family practice, Israel | 11 family practice clinics in Central Israel. | Survey design | To explore the attitudes of patients treated by a first year resident | All patients above 16 years visiting a family clinic on one day. First-time visitors and patients with dementia or mental disability were excluded | 422 eligible patients; 304 participated (72%) | First year residents at least 2 months at the practice | Not specified |
| **Norris & Flaherty et al., 1993** | Family practice, USA | 7 (6 rural, 1 urban setting) family practice clinics affiliated with the Montana Family Residency Program | Survey design | Comparison of patient satisfaction with residents and attending physician | All patients seen by a resident, attending physician or both | 750 eligible patients; 178 participated (23.7%) | Second or third year residents | Not specified |
| **Reichgott & Schwartzet al., 1983** | Internal Medicine, USA | An internal medicine clinic, University of Pennsylvania | Survey design | Evaluation of the attitudes toward resident participation | Private patients who had not yet had a resident involved in their care | 677 eligible patients; 195 participated(29%) | Not specified | Not specified |
| **Rifkin et all., 2002** | Internal medicine, USA | A Health Office and a Planned Parenthood office in Denver, Colorado | Survey design | To discover the characteristics of women who refuse male residents and to describe their reasons for refusing | All women entering the offices over two months | 1437 eligible; 1071 (74%) participated | Not specified | Not specified |
| **Rodney et al., 1986** | Family medicine, USA | 3 family health centers within the San Bernadino County Medical Center Family Practice Program | Survey design | Determine patient satisfaction with trainees, compared to faculty | Patients who had visited the same doctor at least five times in the last two years | 166 eligible patients; 153 (92%) participated | First, second and third-year residents | Not specified |
| **Rodriques et al., 2023** | Family medicine, Canada | 9 family medicine clinics affiliated with the University of Montreal or the McGill University | Survey design | To compare the experience of timely access by patients of residents versus staff, and to determine if there was a difference between both groups in reported appropriateness and patient-centeredness of the visit | All visiting patients in 2018 who were above 18, could read English, were registered I the clinic and who visited before | 1979 included | Not specified | Not specified |
| **Sheets et al., 1991** | Gynecology, USA | Ambulatory care clinic affiliated to an academic center | Survey design | Assessment of the impact of residents on patient satisfaction | All patients seen by resident or by a physician alone over five months | 461 eligible patients; 245 participated (55%) | Second-year residents | Reactive supervision; double-checking after consult |
| **Sherbuk & Barakat et al., 2019** | HIV outpatient clinic, USA | HIV outpatient clinic affiliated to an urban academic medical center | Survey design | Evaluate whether provider level, either trainee or an experienced HIV specialist, affected patient satisfaction. | All patients visiting the clinic between May and October, who are above 18 years of age and with 2 prior visits to their PCP | 89 eligible patients; 75 participated (84%) | Not reported | Not reported |
| **Thornet et al, 2001** | General practice, UK | 1 general practice in Nottinghamshire | Survey design | Comparison of patient satisfaction with residents vs their trainers | All patients visiting the practice until 75 questionnaires for each doctor were completed | 150 participants | First year resident | Not specified |
| **Yancy et al., 2001** | Internal medicine, USA | 4 internal medicine clinics; 2 university-based clinics and 2 veterans’ affairs clinics in Pittsburg | Survey design | Comparison of overall patient satisfaction with residents vs attending physicians | All patients seen by a resident or an attending physician. First-time visitors were excluded | 431 eligible patients; 288 participated (67%) | Second and third-year residents | Not specified |
|  |  |  |  |  |  |  |  |  |
