# Supplementary material for: Patient satisfaction and willingness as indicators for patient perspectives toward trainee involvement: a systematic review
Source: BMC Med Educ. 2025 Dec 22;25:1749. doi: 10.1186/s12909-025-08310-4 (PMC12751747; doi:10.1186/s12909-025-08310-4)
Supplement: Supplementary file 3 — Supplementary Material 3. [file 12909_2025_8310_MOESM3_ESM.docx]

**Appendix III – Quality assessment**

**Section A: MERSQI scores**

| **MERSQI**  **Study** | **Study design (max 3)** | **Sampling**  **(max 3)** | **Type of data**  **(max 3)** | **Validity evidence for evaluation instrument scores (max 3)** | **Data analysis**  **(max 3)** | **Outcome**  **(max 3)** | **Total score**  **(max 18)** |
| --- | --- | --- | --- | --- | --- | --- | --- |
| **AlGhamdi et al., 2014** | 1 | 2 | 1 | 1 | 3 | 1 | 9 |
| **Allen & Bahrami., 1981** | 1 | 1 | 1 | 0 | 2 | 1 | 6 |
| **Bain & Mackay., 1995** | 1 | 3 | 1 | 0 | 2 | 1 | 8 |
| **Blanchard et al., 1977** | 1 | 1 | 1 | 0 | 2 | 1 | 6 |
| **Bonds et al., 2004** | 1 | 2 | 1 | 2 | 3 | 1 | 10 |
| **Bonney et al., 2010** | 1 | 2 | 1 | 2 | 3 | 1 | 10 |
| **Bonney et al., 2012 & Bonney et al., 20 14** | 1 | 2 | 1 | 2 | 3 | 1 | 10 |
| **Bradley et al., 1981** | 1.5 | 1.5 | 1 | 0 | 3 | 1 | 8 |
| **Brahmania et al., 2015** | 1 | 1 | 1 | 2 | 3 | 1 | 9 |
| **Chambers et al., 2022** | 1 | 1 | 1 | 1 | 1 | 1 | 7 |
| **Crawford et al., 2005** | 1 | 2 | 1 | 0 | 3 | 1 | 8 |
| **Cao & Chen et al., 2018** | 1 | 1.5 | 1 | 1 | 3 | 1 | 8.5 |
| **Carruthers et al., 2015** | 1 | 1 | 1 | 1 | 3 | 1 | 8 |
| **Faugeroux et al., 2023** | 1 | 1.5 | 1 | 1 | 3 | 1 | 8.5 |
| **Griffith et al., 2023** | 1 | 1 | 1 | 2 | 3 | 1 | 9 |
| **Haider et al., 2009** | 1 | 1 | 1 | 0 | 2 | 1 | 6 |
| **Heathcote et al., 2007 & Heathcote et al, 2008** | 1 | 2 | 1 | 1 | 2 | 1 | 8 |
| **Jaturapatporn & Dellow et al., 2007** | 1 | 1.5 | 1 | 3 | 3 | 1 | 10.5 |
| **Labgaa et al., 2014** | 1 | 1 | 1 | 2 | 3 | 1 | 9 |
| **Li et al., 2017** | 1 | 1 | 1 | 1 | 3 | 1 | 8 |
| **Malcolm et al., 2008** | 1 | 3 | 1 | 1 | 3 | 1 | 10 |
| **Mantica et al., 2022** | 1 | 2 | 1 | 1 | 3 | 1 | 9 |
| **Murphyet al., 1995** | 1 | 3 | 1 | 1 | 3 | 1 | 10 |
| **Monk et al., 2006** | 1 | 1 | 1 | 1 | 2 | 1 | 7 |
| **Nakar et al., 2010** | 1 | 2.5 | 1 | 1 | 3 | 1 | 9.5 |
| **Norris & Flaherty et al., 1993** | 1 | 1 | 1 | 2 | 2 | 1 | 8 |
| **Reichgott & Schwartzet al., 1983** | 1 | 1 | 1 | 0 | 3 | 1 | 7 |
| **Rifkin et all, 2002** | 1 | 2.5 | 1 | 2 | 3 | 1 | 10.5 |
| **Rodney et al., 1986** | 1 | 2 | 1 | 3 | 3 | 1 | 11 |
| **Rodriques et al., 2023** | 1 | 2 | 1 | 1 | 3 | 1 | 8 |
| **Sheets et al., 1991** | 1 | 1.5 | 1 | 2 | 3 | 1 | 9.5 |
| **Sherbuk & Barakat et al., 2019** | 1 | 2 | 1 | 1 | 3 | 3 | 11 |
| **Thornettet al, 2001** | 1 | 1 | 1 | 2 | 2 | 1 | 8 |
| **Yancy et al., 2001** | 1 | 2 | 1 | 3 | 3 | 1 | 11 |

**Section B: COREQ – assessment qualitative studies (n=2)**

**Study: Bonney et al., 2009**

| **No. Item** | **Guide questions/description** | **Reported on Page #** |
| --- | --- | --- |
| **Domain 1: Research team and reﬂexivity** |  |  |
| *Personal Characteristics* |  |  |
| 1. Interviewer/facilitator | Which author/s conducted the interview or focus group? | AB or LP (page 928) |
| 2. Credentials | What were the researcher’s credentials? E.g. PhD, MD | AB is a GP, LP is an associate research fellow |
|  |  |  |
| 3. Occupation | What was their occupation at the time of the study? | Senior lecture and research fellow |
| 4. Gender | Was the researcher male or female? | Female and male |
| 5. Experience and | What experience or training did the | Not reported |
| training | researcher have? |  |
| *Relationship with participants* |  |  |
| 6. Relationship established | Was a relationship established prior to study commencement? | No |
| 7. Participant knowledge of the interviewer | What did the participants know about  the researcher? e.g. personal goals, reasons for doing the research | Information pack to eligible patients (methods) |
| 8. Interviewer characteristics | What characteristics were reported about the interviewer/facilitator? e.g. Bias, assumptions, reasons and  interests in the research topic | None |
| **Domain 2: study design** |  |  |
| *Theoretical framework* |  |  |
| 9. Methodological orientation and Theory | What methodological orientation was stated to underpin the study? e.g. grounded theory, discourse analysis,  ethnography, phenomenology, content analysis | Template approach (methods) |
| *Participant selection* |  |  |
| 10. Sampling | How were participants selected? e.g.  purposive, convenience, consecutive, snowball | Purposive sampling on gender and previous visits to the trainee (methods) |
| 11. Method of approach | How were participants approached?  e.g. face-to-face, telephone, mail, email | Firstly by the medical practice staff, interested patients were contacted by the research staff. Not clear how they were contacted (methods) |
| 12. Sample size | How many participants were in the study? | 38 interviews (results) |
| 13. Non-participation | How many people refused to participate or dropped out? Reasons? | Not clearly reported in results |
| *Setting* |  |  |
| 14. Setting of data collection | Where was the data collected? e.g. home, clinic, workplace | Not reported |
| 15. Presence of non- participants | Was anyone else present besides the participants and researchers? | Not reported |
| 16. Description of sample | What are the important characteristics of the sample? e.g. demographic data, date | Only gender and age are described (results) |
| *Data collection* |  |  |
| 17. Interview guide | Were questions, prompts, guides provided by the authors? Was it pilot tested? | An interview guide was used based on a literature search (methods) |
| 18. Repeat interviews | Were repeat inter views carried out? If yes, how many? | No |
| 19. Audio/visual recording | Did the research use audio or visual recording to collect the data? | Audio recording (methods) |
| 20. Field notes | Were ﬁeld notes made during and/or after the interview or focus group? | Not reported |
| 21. Duration | What was the duration of the interviews or focus group? | Between 15 and 20 minutes (methods) |
| 22. Data saturation | Was data saturation discussed? | Not reported |
| 23. Transcripts returned | Were transcripts returned to participants for comment and/or | Not reported |
| **Domain 3: analysis and ﬁndings** |  |  |
| *Data analysis* |  |  |
| 24. Number of data coders | How many data coders coded the data? | One author (methods) |
| 25. Description of the  coding tree | Did authors provide a description of  the coding tree? | Not reported |
| 26. Derivation of themes | Were themes identiﬁed in advance or derived from the data? | Derived from data (methods) |
| 27. Software | What software, if applicable, was used  to manage the data? | Not reported |
| 28. Participant checking | Did participants provide feedback on the ﬁndings? | Not reported |
| *Reporting* |  |  |
| 29. Quotations presented | Were participant quotations presented to illustrate the themes/ﬁndings? Was each quotation identiﬁed? e.g.  participant number | Yes (results) |
| 30. Data and ﬁndings consistent | Was there consistency between the data presented and the ﬁndings? | Yes (discussion) |
| 31. Clarity of major themes | Were major themes clearly presented in the ﬁndings? | Yes (results) |
| 32. Clarity of minor themes | Is there a description of diverse cases or discussion of minor themes? | Yes (results) |

**Study: De Bever et al., 2022**

| **No. Item** | **Guide questions/description** | **Reported on Page #** |
| --- | --- | --- |
| **Domain 1: Research team and reﬂexivity** |  |  |
| *Personal Characteristics* |  |  |
| 1. Interviewer/facilitator | Which author/s conducted the interview or focus group? | The interviews were conducted by SvR, MV and SdB (Page 2) |
| 2. Credentials | What were the researcher’s credentials? E.g. PhD, MD | The diversity of this team ranged from people working in GP practices (SdB, JB, AK) to people more distant from practices (MV, NvD, SvR) and with educational research experience (MV, NvD, AK, SvR) ensured a broad range of perspectives on the data. (Page 3) |
|  |  |  |
| 3. Occupation | What was their occupation at the time of the study? | See above |
| 4. Gender | Was the researcher male or female? | Not reported |
| 5. Experience and | What experience or training did the | See above |
| training | researcher have? |  |
| *Relationship with participants* |  |  |
| 6. Relationship established | Was a relationship established prior to study commencement? | Not reported |
| 7. Participant knowledge of the interviewer | What did the participants know about  the researcher? e.g. personal goals, reasons for doing the research | Patients were given information about the study (page 3) |
| 8. Interviewer characteristics | What characteristics were reported about the interviewer/facilitator? e.g. Bias, assumptions, reasons and  interests in the research topic | Not reported |
| **Domain 2: study design** |  |  |
| *Theoretical framework* |  |  |
| 9. Methodological orientation and Theory | What methodological orientation was stated to underpin the study? e.g. grounded theory, discourse analysis,  ethnography, phenomenology, content analysis | We used thematic analysis, with a constant comparative approach. Page 2 |
| *Participant selection* |  |  |
| 10. Sampling | How were participants selected? e.g.  purposive, convenience, consecutive, snowball | Patients were purposively sampled to gain maximum variation in willingness to consult GP trainees. Page 2 |
| 11. Method of approach | How were participants approached?  e.g. face-to-face, telephone, mail, email | Unwilling patients were selected by the medical receptionist or GP, and were sent an information letter and application form. Willing patients were recruited after their visit to the trainee. Page 2 |
| 12. Sample size | How many participants were in the study? | We held 28 interviews. Page 3 |
| 13. Non-participation | How many people refused to participate or dropped out? Reasons? | Not reported |
| *Setting* |  |  |
| 14. Setting of data collection | Where was the data collected? e.g. home, clinic, workplace | At a place f preference for the unwilling patients and in the GP office for the willing patients Page 2 |
| 15. Presence of non- participants | Was anyone else present besides the participants and researchers? | Not reported |
| 16. Description of sample | What are the important characteristics of the sample? e.g. demographic data, date | More females than males participated and unwilling patients are older than willing ones. Page 3 |
| *Data collection* |  |  |
| 17. Interview guide | Were questions, prompts, guides provided by the authors? Was it pilot tested? | Semi-structured interviews were held using a topic guide. Page 2 |
| 18. Repeat interviews | Were repeat inter views carried out? If yes, how many? | Not reported |
| 19. Audio/visual recording | Did the research use audio or visual recording to collect the data? | Used an audio recorder. Page 2 |
| 20. Field notes | Were ﬁeld notes made during and/or after the interview or focus group? | Yes Page 2 |
| 21. Duration | What was the duration of the interviews or focus group? | Interviews lasted between 6 and 31 minutes. Page 3 |
| 22. Data saturation | Was data saturation discussed? | Yes. Page 3 |
| 23. Transcripts returned | Were transcripts returned to participants for comment and/or | Not reported |
| **Domain 3: analysis and ﬁndings** |  |  |
| *Data analysis* |  |  |
| 24. Number of data coders | How many data coders coded the data? | In total 3. Page 2 |
| 25. Description of the  coding tree | Did authors provide a description of  the coding tree? | Yes Page 3 |
| 26. Derivation of themes | Were themes identiﬁed in advance or derived from the data? | Derived from the data. Page 3 |
| 27. Software | What software, if applicable, was used  to manage the data? | MaxQDA Page 3 |
| 28. Participant checking | Did participants provide feedback on the ﬁndings? | Not reported |
| *Reporting* |  |  |
| 29. Quotations presented | Were participant quotations presented to illustrate the themes/ﬁndings? Was each quotation identiﬁed? e.g.  participant number | Yes. Page 3-5 |
| 30. Data and ﬁndings consistent | Was there consistency between the data presented and the ﬁndings? | Yes. Page 3-5 |
| 31. Clarity of major themes | Were major themes clearly presented in the ﬁndings? | Yes. Page 3-5 |
| 32. Clarity of minor themes | Is there a description of diverse cases or discussion of minor themes? | Yes. Page 3-5 |
