# Supplementary material for: Patient satisfaction and willingness as indicators for patient perspectives toward trainee involvement: a systematic review
Source: BMC Med Educ. 2025 Dec 22;25:1749. doi: 10.1186/s12909-025-08310-4 (PMC12751747; doi:10.1186/s12909-025-08310-4)
Supplement: Supplementary file 4 — Supplementary Material 4. [file 12909_2025_8310_MOESM4_ESM.docx]

| **Study** | **Reason for exclusion** |
| --- | --- |
| Agarwal et al., 2020(1) | Design |
| Al-Harbi et al., 2010(2) | Population |
| Ali et al., 2013(3) | Design |
| Alpert et al., 1975(4) | Design |
| Anonymous., 2013(5) | Design |
| Ashburner et al., 2013(6) | Design |
| Aunsholt et al., 2008(7) | Language |
| Baghus et al., 2024(8) | Outcome |
| Baker et al., 1996(9) | Outcome |
| Bartlett et al ., 1984(10) | Outcome |
| Bertakis et al ., 1995(11) | Outcome |
| Bestvater et al., 1988(12) | Outcome |
| Blanco Canseco et al., 2018(13) | Language |
| Bonney et a;., 2015(14) | Outcome |
| Bornstein et al., 2000(15) | Outcome |
| Boutin-Foster et al., 2002(16) | Outcome |
| Bradford et al., 1994(17) | Outcome |
| Brook et al., 1987(18) | Outcome |
| Brown et al., 1997(19) | Outcome |
| Caballero Jeuregui et al., 2008(20) | Language |
| Chen et al., 2010(21) | Design |
| Curtin et al., 1986(22) | Design |
| Dambha et al., 2014(23) | Design |
| Drevs et al., 2014(24) | Outcome |
| Dutta et al., 2003(25) | Population |
| El-Sayeh et al., 2003(26) | Design |
| Ezra et al., 2009(27) | Population |
| Falvo et al., 1983(28) | Outcome |
| Fayaz-Bakhsh et al., 2016(29) | Design |
| Feddock et al., 2015(30) | Outcome |
| Fiebach et al., 2001(31) | Design |
| Fuglsang et al., 1996(32) | Language |
| Gerace et al., 1987(33) | Outcome |
| Gilbert et al., 2004(34) | Design |
| Gill et al., 2023(35) | Population |
| Gross et al., 1998(36) | Outcome |
| Guffey et al., 2009(37) | Outcome |
| Hess et al., 2011(38) | Outcome |
| Hoellein et al., 2014(39) | Outcome |
| Hunt et al., 1977(40) | Population |
| Husk et al., 2013(41) | Design |
| Huynh et al., 2012(42) | Outcome |
| Jackson et al., 1999(43) | Outcome |
| Jalil et al., 2017(44) | Outcome |
| Jenkins et al., 1996(45) | Outcome |
| Kamanger er al., 2016(46) | Population |
| Kempenich et al., 2016(47) | Outcome |
| Kempenich et al., 2018(48) | Outcome |
| Kojima et al., 2022(49) | Population |
| Kostov et al., 2017(50) | Population |
| Krol et al., 1983(51) | Outcome |
| Kuraitis et al., 2022(52) | Population |
| Lehmann et al., 1988(53) | Outcome |
| Linn et al., 1984(54) | Outcome |
| Long et al., 2016(55) | Outcome |
| Lynn et al., 2012(56) | Outcome |
| Miller er al., 2012(57) | Design |
| Morrison et al., 2014(58) | Outcome |
| Murphy-Cullen et al., 1984(59) | Outcome |
| Nouri et al., 2017(60) | Population |
| O’Malley et al., 1997(61) | Population |
| Probst et al., 1997(62) | Outcome |
| Qidwai et al., 2003(63) | Outcome |
| Rabanaque et al., 2005(64) | Language |
| Reid et al., 2014(65) | Outcome |
| Robinson et al., 2008(66) | Design |
| Sakti et al., 2022(67) | Outcome |
| Smith et al., 1995(68) | Outcome |
| Steinbook et al., 2007(69) | Outcome |
| Swor et al., 2014(70) | Design |
| Tombesi et al., 1995(71) | Language |
| Treadway et al., 1983(72) | Outcome |
| Unruh et al., 2013 (73) | Outcome |
| Yab et al., 2019(74) | Outcome |
| Zeller et al., 2006(75) | Outcome |
| Zhu et al., 2016(76) | Population |

**References**

1. Agarwal Y, Beatty C, Biradar S, Castronova I, Ho S, Melody K, et al. Moving bey

ond the mousetrap: current and emerging humanized mouse and rat models for investigating prevention and cure strategies against HIV infection and associated pathologies. Retrovirology. 2020;17(1):8.

2. Al-Harbi M, Al-Harbi K. Patient Attitudes towards Interns and Medical Students rotating in Dermatology Clinics in Almadinah Almunawwarah Region, Saudi Arabia. Sultan Qaboos University Medical Journal. 2010;10(3):377-81.

3. Ali K, Shayne P, Ross M, Franks N. Evaluation of the patient satisfaction performance of emergency medicine resident physicians in a large urban academic emergency department. Annals of Emergency Medicine. 2013;1):S139.

4. Alpert JJ. Graduate Education for Primary Care: Problems and Issues. Journal of Medical Education. 1975;50(12):123-8.

5. Anonymous. Council of Emergency Medicine Residency Directors, CORD Academic Assembly 2013. Annals of Emergency Medicine Conference: Council of Emergency Medicine Residency Directors, CORD Academic Assembly. 2013;62(5).

6. Ashburner JM, Ward CE, Chang Y, Fosburgh BW, Atlas SJ. Differences in patient perceptions of the quality of communication among staff and resident primary care physicians. Journal of General Internal Medicine. 2012;27:S162-S3.

7. Aunsholt L, Ammentorp J, Jorgensen S, Kofoed PE. [Evaluation of satisfaction with training in tuition outpatient clinic]. Ugeskrift for Laeger. 2008;170(45):3654-9.

8. Baghus A, Giroldi E, van Geel J, Leferink A, van de Pol MHJ, Sanders A, et al. Shared decision-making performance of general practice residents: an observational study combining observer, resident, and patient perspectives. Fam Pract. 2024;41(1):50-9.

9. Baker R. Characteristics of practices, general practitioners and patients related to levels of patients' satisfaction with consultations. British Journal of General Practice. 1996;46(411):601-5.

10. Bartlett EE, Grayson M, Barker R. The effects of physician communications skills on patient satisfaction; recall, and adherence. Journal of Chronic Diseases. 1984;37(9):755-64.

11. Bertakis KD, Helms LJ, Callahan EJ, Azari R, Robbins JA. The influence of gender on physician practice style. Medical care.33(4):407-16.

12. Bestvater D, Dunn EV, Nelson W, Townsend C. The effects of learners on waiting times and patient satisfaction in an ambulatory teaching practice. Family Medicine. 1988;20(1):39-42.

13. Blanco Canseco JM, Valcarcel Sierra C, Guerra Jimenez MDC, Ortigosa Rodriguez E, Garcia Lopez F, Caballero Martinez F, et al. Clinical empathy among family and community medicine residents and tutors. The view of physicians and patients. Atencion Primaria. 2018.

14. Bonney A, Phillipson L, Jones SC, Hall J, Sharma R. The brave new world of older patients: preparing general practice training for an ageing population. Primary Health Care Research & Development. 2015;16(6):578-88.

15. Bornstein BH, Marcus D, Cassidy W. Choosing a doctor: an exploratory study of factors influencing patients' choice of a primary care doctor. Journal of Evaluation in Clinical Practice. 2000;6(3):255-62.

16. Boutin-Foster C, Charlson ME. Problematic resident-patient relationships: The patient's perspective. Journal of General Internal Medicine.16(11):750-4.

17. Bradford BJ, Benedum K, Heald PA. The prenatal pediatric visit and pediatric residency training. Clinical Pediatrics. 1994;33(11):688-90.

18. Brook RH, Fink A, Kosecoff J, Linn LS, Watson WE, Davies AR, et al. Educating physicians and treating patients in the ambulatory setting. Where are we going and how will we know when we arrive? Annals of Internal Medicine. 1987;107(3):392-8.

19. Brown JB, Dickie I, Brown L, Biehn J. Long-term attendance at a family practice teaching unit. Qualitative study of patients' views. Canadian Family Physician. 1997;43:901-6.

20. Caballero Jauregui M, Salamanca Sanchez-Escalonilla MT, Saniger Herrera JM, Munoz Gonzalez F, Miguel Calvo I, Garcia Ledesma L. [Do our patients know what a family medicine resident is?]. Atencion Primaria. 2008;40(1):15-9.

21. Chen J, McPherson-Albers U, Fontanesi J. Resolving competing priorities in resident clinic. Journal of General Internal Medicine. 2010;25:S470.

22. Curtin SF. World view and the biopsychosocial model of medicine: Medical resident behavior and patient satisfaction. Dissertation Abstracts International.46(5):1729.

23. Dambha H. Patient experience and GP trainees. The British journal of general practice : the journal of the Royal College of General Practitioners. 2014;64(622):222.

24. Drevs F, Gebele C, Tscheulin DK. The patient perspective of clinical training-An empirical study about patient motives to participate. Health Policy. 2014;118(1):74-83.

25. Dutta S, Dunnington G, Blanchard MC, Spielman B, DaRosa D, Joehl RJ. And doctor, no residents please! Journal of the American College of Surgeons. 2003;197(6):1012-7.

26. El-Sayeh HG. Using student doctor or trainee doctor may be helpful [6]. British Medical Journal. 2003;327(7423):1110.

27. Ezra DG, Salam T, Sullivan PM, Okhravi N. Towards a better understanding of patient perspectives of clinical teaching in ophthalmology. Eye. 2009;23(8):1681-4.

28. Falvo DR, Smith JK. Assessing residents' behavioral science skills: Patients' views of physician-patient interaction. The Journal of family practice. 1983;17(3):479-83.

29. Fayaz-Bakhsh A, Nasiri T. Physician Identification and Patient Satisfaction: Are They Related? Journal of Emergency Medicine.50(3):e175.

30. Feddock CA, Hoellein AR, Griffith CH, Wilson JF, Becker NS, Bowerman JL, et al. Are continuity clinic patients less satisfied when residents have a heavy inpatient workload? Evaluation and the Health Professions.28(4):390-9.

31. Fiebach NH, Wong JG. Taking care of patients in resident clinics: Where do we stand? Journal of General Internal Medicine. 2001;16(11):787-9.

32. Fuglsang H, Olesgaard P, Pedersen NF, Olesen F. [Patients' attitudes towards and satisfaction with interns in general practice. Practicing interns and patient satisfaction]. Ugeskrift for Laeger. 1996;158(41):5768-72.

33. Gerace TM, Sangster JF. Factors determining patients' satisfaction in a family practice residency teaching center. Journal of Medical Education. 1987;62(6):485-90.

34. Gilbert HC, Rich BA, Fine P. Quality of care, teaching responsibilities, and patient's preferences. Pain Medicine.5(2):206-11.

35. Gill J, Hawkins D, Carrico C, Laskin DM. How do patients perceive resident participation in facial cosmetic treatment? Oral Surg Oral Med Oral Pathol Oral Radiol. 2023;136(2):142-6.

36. Gross C, Callahan M, Mele J. Will commercial managed care patients accept residents as their primary care providers? Journal of General Internal Medicine. 1998;13(5):331-4.

37. Guffey R, Juzych N, Juzych M. Patient knowledge of physician responsibilities and their preferences for care in ophthalmology teaching clinics. Ophthalmology. 2009;116(9):1610-4.

38. Hess BJ, Lynn LA, Conforti LN, Holmboe ES. Listening to older adults: elderly patients' experience of care in residency and practicing physician outpatient clinics. Journal of the American Geriatrics Society. 2011;59(5):909-15.

39. Hoellein AR, Feddock CA, Griffith CH, 3rd, Wilson JF, Barnett DR, Bass PF, 3rd, et al. Are continuity clinic patients less satisfied when the resident is postcall? Journal of General Internal Medicine. 2004;19(5):562-5.

40. Hunt D, Lentz G. Impact of medical student training in psychiatric outpatient settings. Journal of Psychiatric Education.12(3):204-11.

41. Husk G, Wang M, Lowery S, Akhtar S. Are patients treated by residents as satisfied as those treated by mid level providers or attending physicians? Annals of Emergency Medicine. 2013;62 (5):S168.

42. Huynh M, Lee AD, Miller LM, Davis S, Feldman SR, McMichael A. Patients' satisfaction with dermatology residents. Southern Medical Journal. 2012;105(10):520-3.

43. Jackson JL, Kroenke K, Pangaro L. A comparison of outcomes for walk-in clinic patients who see interns and those who see staff physicians. Academic Medicine. 1999;74(6):718-20.

44. Jalil A, Zakar R, Zakar MZ, Fischer F. Patient satisfaction with doctor-patient interactions: a mixed methods study among diabetes mellitus patients in Pakistan. BMC health services research.17(1):155.

45. Jenkins M, Thomas A. The assessment of general practitioner registrars' consultations by a patient satisfaction questionnaire. Medical Teacher. 1996;18(4):347-50.

46. Kamangar F, Davari P, Parsi KK, Li CS, Wang Q, Mathis S, et al. 360-degree evaluations on physician performance as an effective tool for interprofessional teams: A critical analysis of physician self-assessment as compared to nursing staff and patient evaluations of providers. Dermatology Online Journal. 2016;22(7).

47. Kempenich JW, Willis RE, Blue RJ, Al Fayyadh MJ, Cromer RM, Schenarts PJ, et al. The Effect of Patient Education on the Perceptions of Resident Participation in Surgical Care. Journal of Surgical Education. 2016;73(6):e111-e7.

48. Kempenich JW, Willis RE, Fayyadh MA, Campi HD, Cardenas T, Hopper WA, et al. Video-Based Patient Education Improves Patient Attitudes Toward Resident Participation in Outpatient Surgical Care. Journal of Surgical Education. 2018;75(6):e61-e7.

49. Kojima L, Han AY, French JC, Lipman JM. The Patient's Voice in Assessing Resident Communication Skills. J Surg Educ. 2022;79(6):e220-e4.

50. Kostov CE, Rees CE, Gormley GJ, Monrouxe LV. I did try and point out about his dignity ': A qualitative narrative study of patients and carers' experiences and expectations of junior doctors. BMJ Open.8.

51. Krol RA, Nordlund DJ. Patient-satisfaction data and residents' physician-patient skills. The Journal of family practice. 1983;17(1):141-2.

52. Kuraitis D, Murina A. Gender discordance of genital examination experiences among dermatology residents and attendings. J Am Acad Dermatol. 2022;86(3):638-40.

53. Lehmann F, Fontaine D, Bourque A, Cote L. Measurement of patient satisfaction: the smith-falvo patient-doctor interaction scale. Canadian Family Physician. 1988;34:2641-5.

54. Linn LS, al. e. The Effect of Gender and Training of Residents on Satisfaction Ratings by Patients. Journal of Medical Education. 1984;59(12):964-66.

55. Long J, Morton L, Taylor E. What Not to Wear: An Analysis of Outpatient Resident Attire. Academic psychiatry : the journal of the American Association of Directors of Psychiatric Residency Training and the Association for Academic Psychiatry.41(3):411-6.

56. Lynn L, Hess BJ, Weng W, Lipner RS, Holmboe ES. Gaps in quality of diabetes care in internal medicine residency clinics suggest the need for better ambulatory care training. Health Affairs.31(1):150-8.

57. Miller MP, Chacko B, Nugent A, Harland K, Denning G. Type and level of resident education affects emergency department patient satisfaction. Annals of Emergency Medicine. 2012;1):S149.

58. Morrison T, Brown J, Bryant M, Nestel D. Benefits and challenges of multi-level learner rural general practices--an interview study with learners, staff and patients. BMC medical education. 2014;14:234.

59. Murphy-Cullen CL, Larsen LC. Interaction between the socio-demographic variables of physicians and their patients: its impact upon patient satisfaction. Social Science & Medicine. 1984;19(2):163-6.

60. Nouri M, Ghaffarifar S, Bazargani HS, Ghaffari R. Patients' satisfaction with medical residents'communication skills at the largest teaching and treatment center in North West Iran in 2016. Shiraz E Medical Journal. 2017;18.

61. O'Malley PG, Omori DM, L, ry FJ, Jackson J, Kronke K. A prospective study to assess the effect of ambulatory teaching on patient satisfaction. Academic Medicine.72(11):1015-7.

62. Probst JC, Greenhouse DL, Selassie AW. Patient and physician satisfaction with an outpatient care visit. Journal of Family Practice.45(5):418-25.

63. Qidwai W, Dhanani RH, Khan FM. Implications for the practice of a patient expectation and satisfaction survey, at a teaching hospital in Karachi, Pakistan. JPMA - Journal of the Pakistan Medical Association. 2003;53(3):122-5.

64. Rabanaque Mallen G, Garcia Domingo C, Martinez Perpina S, Dolz Domingo A, Ordono Dominguez F, Rubio Sanjaime P. What are users' views of medical residents attending primary care consultations?. [Spanish]. Atencion Primaria. 2005;36(1):25-30.

65. Reid DR, Makinde KA, Wilson MP, PausJenssen AM, Barton JW, Wilson TW. Dress and deportment of medical residents: formal or informal? Clinical & Investigative Medicine - Medecine Clinique et Experimentale. 2014;37(4):E258-61.

66. Robinson BL, Saks EK, Gopal M, Harvie H, Ronner W, Aryat L. Willingness of gynecologic patients to be interviewed and examined by female and male medical students and resident physicians. Journal of Pelvic Medicine and Surgery. 2008;14 (4):243.

67. Sakti DH, Firdaus AT, Utami TP, Jati KDP, Mahayana IT, Wardhana FS, et al. Patients' Satisfaction with Ophthalmology Clinic Services in a Public Teaching Hospital. Patient Prefer Adherence. 2022;16:723-35.

68. Smith RC, Lyles JS, Mettler JA, Marshall AA, Van Egeren L, Stoffelmayr B, et al. A strategy for improving patient satisfaction by the intensive training of residents in psychosocial medicine: A controlled, randomized study. Academic Medicine.70(8):729-32.

69. Steinbook RM. Continuity clinics in psychiatric residency training. Academic Psychiatry.31(1):15-8.

70. Swor R, Ziadeh J, Jayasankar S. Comparison of attending-only and resident with attending supervision press ganey scores in ED patients. Academic Emergency Medicine. 2014;1):S218.

71. Tombesi M. A young trainee in the GP's office: What do patients think?. [Italian]. Ricerca e Pratica. 1995(63):107-14.

72. Treadway J. Patient satisfaction and the content of general practice consultations. Journal of the Royal College of General Practitioners. 1983;33(257):769-71.

73. Unruh KP, Dhulipala SC, Holt GE. Patient understanding of the role of the orthopedic resident. Journal of Surgical Education. 2013;70(3):345-9.

74. Yab J. Attitudes and perspectives of patients toward medical trainees. Proceedings in Singapore Healthcare; 2019;28(2):129-30.

75. Zeller M, Perruzza E, Austin L, Vohra S, Stephens D, Abdolell M, et al. Parental understanding of the role of trainees in the ophthalmic care of their children. Ophthalmology. 2006;113(12):2292-7.

76. Zhu Y, Yan T, Qu B. Assessment of Surgery Resident Competency Provided by Patients. Journal of Craniofacial Surgery. 2016;27(8):2105-9.

a
